# Supplementary material for: Transcutaneous spinal cord stimulation and motor responses in individuals with spinal cord injury: A methodological review
Source: PLoS One. 2021 Nov 18;16(11):e0260166. doi: 10.1371/journal.pone.0260166 (PMC8601579; doi:10.1371/journal.pone.0260166)
Supplement: S1 Appendix — (DOCX) [file pone.0260166.s002.docx]

**S1 Appendix - Search Strategy and Terminology**

**Final Search Date: 30/06/20**

**EMBASE 1739 Articles**

1. 'spinal cord stimulation'/exp

2. ((Spinal OR ‘Dorsal Root’ OR ‘Posterior Root’ OR spine OR non-invasive) NEAR/3 stimulation):ti,ab

3. ((intraspinal OR intra-spinal OR transpinal OR transspinal OR 'trans spinal') NEAR/3 stimulation):ti,ab

4. (‘Spinal modulation’ OR neuromodulation OR ‘Altering spinal’ OR ‘Altering spinal excitability’):ti,ab

5. ((Epidural) NEAR/3 (electrostimulat* OR stimulat*)):ti,ab

6. #1 OR #2 OR #3 OR #4 OR #5

7. 'motor evoked potential'/exp OR 'evoked spinal cord response'/exp OR 'monosynaptic pathway'/exp OR 'voluntary movement'/exp OR 'muscle contraction'/exp OR 'motor performance'/exp OR 'locomotion'/exp

8. ((motor OR locomotor OR locomotion) NEAR/3 (function* OR reflex OR response* OR potential OR performance OR train*)):ti,ab

9. ((muscle* OR muscular) NEAR/4 (function* OR activat* OR reflex* OR response* OR contract*)):ti,ab

10. (Limb* NEAR/3 movement*):ti,ab

11. ('transspinal evoked potential' OR ‘posterior root reflex*’ OR ‘sensorimotor network functionality’ OR ‘monosynaptic response*’ OR ‘multisegmental response’ OR ‘circuitry output*’ OR ‘voluntary movement*’ OR ‘Augmented control strateg*’ OR ‘stepping movement*’ OR ‘Locomotor circuit*’):ti,ab

12. 'electromyogram'/exp

13. (Electromyogra* OR ‘electric myogra*’ OR ‘electrical myogra*’ OR ‘electro myogra*’ OR EMG):ti,ab

14. #7 OR #8 OR #9 OR #10 OR #11 OR #12 OR #13

15. #6 AND #14

16. 'editorial'/exp OR 'erratum'/de OR 'letter'/exp OR 'conference abstract':it OR 'conference review':it OR (('animal'/exp OR 'nonhuman'/exp) NOT (('animal'/exp OR 'nonhuman'/exp) AND 'human'/exp))

17. #15 NOT #16

**Medline (OVID) 1355 Articles**

1. Spinal Cord Stimulation/

2. ((Spinal OR Dorsal Root OR Posterior Root OR spine OR non-invasive) ADJ3 stimulation).ti,ab.

3. ((intraspinal OR intra-spinal OR transpinal OR transspinal OR trans spinal) ADJ3 stimulation).ti,ab.

4. (Spinal modulation OR neuromodulation OR Altering spinal OR Altering spinal excitability).ti,ab.

5. ((Epidural) ADJ3 (electrostimulat* OR stimulat*)).ti,ab.

6. or/1-5

7. exp Evoked Potentials, Motor/ OR exp Reflex, Monosynaptic/ OR exp Muscle Contraction/ OR Motor Activity/ OR exp locomotion/ OR Movement/

8. ((motor OR locomotor OR locomotion) ADJ3 (function* OR reflex OR response* OR potential OR performance OR train*)).ti,ab.

9. ((muscle* OR muscular) ADJ4 (function* OR activat* OR reflex* OR response* OR contract*)).ti,ab.

10. (Limb* ADJ3 movement*).ti,ab.

11. (transspinal evoked potential OR posterior root reflex* OR sensorimotor network functionality OR monosynaptic response* OR multisegmental response OR circuitry output* OR voluntary movement* OR Augmented control strateg* OR stepping movement* OR Locomotor circuit*).ti,ab.

12. electromyogram/

13. (Electromyogra* OR electric myogra* OR electrical myogra* OR electro myogra* OR EMG).ti,ab.

14. or/7-13

15. 6 AND 14

16. (Animals/ or Models, Animal/ or Disease Models, Animal/) not Humans/

17. ((animal or animals or canine* or dog or dogs or feline or hamster* or lamb or lambs or mice or monkey or monkeys or mouse or murine or pig or pigs or piglet* or porcine or primate* or rabbit* or rats or rat or rodent* or sheep* or veterinar*) not (human* or patient*)).ti,kf,jw.

18. or/16-17

19. 15 NOT 18

**Web of Science 341 Articles**

TS =((((Spinal OR “Dorsal Root” OR “Posterior Root” OR spine OR non-invasive) NEAR/2 stimulation) OR ((intraspinal OR intra-spinal OR transpinal OR transspinal OR “trans spinal”) NEAR/2 stimulation) OR (“Spinal modulation” OR neuromodulation OR “Altering spinal” OR “Altering spinal excitability”) OR ((Epidural) NEAR/3 (electrostimulat* OR stimulat*))) AND (((motor OR locomotor OR locomotion) NEAR/2 (function* OR reflex OR response* OR potential OR performance OR train*)) OR ((muscle* OR muscular) NEAR/3 (function* OR activat* OR reflex* OR response* OR contract*)) OR (Limb* NEAR/2 movement*)) AND ((“transspinal evoked potential” OR “posterior root reflex*” OR “sensorimotor network functionality” OR “monosynaptic response*” OR “multisegmental response” OR “circuitry output*” OR “voluntary movement*” OR “Augmented control strateg*” OR “stepping movement*” OR “Locomotor circuit*”) OR (Electromyogra* OR “electric myogra*” OR “electrical myogra*” OR “electro myogra*” OR EMG)))
