# Supplementary material for: Transcutaneous spinal cord stimulation and motor responses in individuals with spinal cord injury: A methodological review
Source: PLoS One. 2021 Nov 18;16(11):e0260166. doi: 10.1371/journal.pone.0260166 (PMC8601579; doi:10.1371/journal.pone.0260166)
Supplement: Attachment — Submitted filename: PONE-D-21-17243_reviewer_comments_highlight.pdf [file pone.0260166.s003.pdf]

PLOS ONE

Transcutaneous spinal cord stimulation and the generation of motor responses in individuals with spinal cord injury: a methodological review

--Manuscript Draft--

**Manuscript Number:**	PONE-D-21-17243
**Article Type:**	Research Article
**Full Title:**	Transcutaneous spinal cord stimulation and the generation of motor responses in individuals with spinal cord injury: a methodological review
**Short Title:**	Transcutaneous spinal cord stimulation and motor responses, a methodological review.
**Corresponding Author:**	Clare Taylor, M.Sc.<br>Trinity College Dublin: The University of Dublin Trinity College<br>Dublin 2, None IRELAND
**Keywords:**	motor activity; neurological rehabilitation; neuromodulation; spinal cord injury; transcutaneous spinal cord stimulation; electromyography; motor evoked potential (MEP); Posterior-root muscle reflex (PRM); multisegmental monosynaptic response (MMR); spinal cord stimulation; spinal reflexes; electrophysiological recording
**Abstract:**	Background: Transcutaneous spinal cord stimulation (tSCS) is a non-invasive modality in which electrodes may stimulate spinal circuitries to produce a motor response. This review aimed to evaluate the methodology of studies using tSCS to generate motor activity in persons with spinal cord injury (SCI) and to appraise the quality of included trials.<br>Methods: A systematic search for studies published until June 2020 was made of the following databases: EMBASE, Medline (Ovid) and Web of Science. Two reviewers independently screened the studies, extracted the data, and evaluated the quality of included trials. The electrical characteristics of stimulation were summarised to allow for comparison across studies. In addition, the electrophysiological recording methods were evaluated.<br>Results : A total of 2222 articles were initially screened, of which 22 met the criteria for inclusion. Studies were divided into those using tSCS for neurophysiological investigations of reflex responses (n = 8) and therapeutic investigations of motor recovery (n = 14). The overall quality of evidence was deemed to be poor-to-fair (9.7 ± 5) based on the Downs and Black Quality Checklist criteria. The methods employed by included studies relating to stimulation parameters and outcome measurement varied extensively, although some trends are emerging in relation to electrode configuration and electromyographic (EMG) outcomes.<br>Conclusion : This review outlines the parameters currently employed for tSCS of the cervicothoracic and thoracolumbar regions to produce motor responses. However, to establish standardised procedures for neurophysiological assessments and therapeutic investigations of tSCS, further high-quality investigations are required, ideally utilizing consistent electrophysiological recording methods, and reporting common characteristics of the electrical stimulation administered.
**Order of Authors:**	Clare Taylor, M.Sc.
	Conor McHugh
	David Mockler
	Conor Minogue
	Richard B. Reilly
	Neil Fleming
**Additional Information:**	
**Question**	**Response**
**Financial Disclosure**	This study was supported by the Disruptive Technology Innovation Fund, grant number DT -2018-0128 (RR, NF, CM)

Powered by Editorial Manager® and ProduXion Manager® from Aries Systems Corporation

Enter a financial disclosure statement that describes the sources of funding for the work included in this submission. Review the submission guidelines for detailed requirements. View published research articles from *PLOS ONE* for specific examples.<br><br>This statement is required for submission and **will appear in the published article** if the submission is accepted. Please make sure it is accurate.<br><br>**Unfunded studies**<br>Enter: *The author(s) received no specific funding for this work.*<br><br>**Funded studies**<br>Enter a statement with the following details:<br>• Initials of the authors who received each award<br>• Grant numbers awarded to each author<br>• The full name of each funder<br>• URL of each funder website<br>• Did the sponsors or funders play any role in the study design, data collection and analysis, decision to publish, or preparation of the manuscript?<br>• **NO** - Include this sentence at the end of your statement: *The funders had no role in study design, data collection and analysis, decision to publish, or preparation of the manuscript.*<br>• **YES** - Specify the role(s) played.<br><br>* typeset	The funders had no role in study design, data collection and analysis, decision to publish, or preparation of the manuscript.
**Competing Interests**<br><br>Use the instructions below to enter a competing interest statement for this submission. On behalf of all authors, disclose any competing interests that could be perceived to bias this work—acknowledging all financial support and any other relevant financial or non-financial competing interests.<br><br>This statement is required for submission and **will appear in the published article** if the submission is accepted. Please make	The authors have declared that no competing interests exist.

sure it is accurate and that any funding sources listed in your Funding Information later in the submission form are also declared in your Financial Disclosure statement.<br><br>View published research articles from *PLOS ONE* for specific examples.<br><br>**NO authors have competing interests**<br><br>Enter: *The authors have declared that no competing interests exist.*<br><br>**Authors with competing interests**<br><br>Enter competing interest details beginning with this statement:<br><br>*I have read the journal's policy and the authors of this manuscript have the following competing interests: [insert competing interests here]*<br><br>* typeset	
**Ethics Statement**<br><br>Enter an ethics statement for this submission. This statement is required if the study involved:<br><br>• Human participants<br>• Human specimens or tissue<br>• Vertebrate animals or cephalopods<br>• Vertebrate embryos or tissues<br>• Field research<br><br>Write "N/A" if the submission does not require an ethics statement.<br><br>General guidance is provided below. Consult the submission guidelines for detailed instructions. **Make sure that all information entered here is included in the Methods section of the manuscript.**	N/A<br>Review article

**Format for specific study types**<br><br>**Human Subject Research (involving human participants and/or tissue)**<br>• Give the name of the institutional review board or ethics committee that approved the study<br>• Include the approval number and/or a statement indicating approval of this research<br>• Indicate the form of consent obtained (written/oral) or the reason that consent was not obtained (e.g. the data were analyzed anonymously)<br><br>**Animal Research (involving vertebrate animals, embryos or tissues)**<br>• Provide the name of the Institutional Animal Care and Use Committee (IACUC) or other relevant ethics board that reviewed the study protocol, and indicate whether they approved this research or granted a formal waiver of ethical approval<br>• Include an approval number if one was obtained<br>• If the study involved *non-human primates*, add *additional details* about animal welfare and steps taken to ameliorate suffering<br>• If anesthesia, euthanasia, or any kind of animal sacrifice is part of the study, include briefly which substances and/or methods were applied<br><br>**Field Research**<br><br>Include the following details if this study involves the collection of plant, animal, or other materials from a natural setting:<br>• Field permit number<br>• Name of the institution or relevant body that granted permission	
**Data Availability**<br><br>Authors are required to make all data underlying the findings described fully available, without restriction, and from the time of publication. PLOS allows rare exceptions to address legal and ethical concerns. See the PLOS Data Policy and FAQ for detailed information.	Yes - all data are fully available without restriction

A Data Availability Statement describing where the data can be found is required at submission. Your answers to this question constitute the Data Availability Statement and **will be published in the article**, if accepted.<br><br>**Important:** Stating 'data available on request from the author' is not sufficient. If your data are only available upon request, select 'No' for the first question and explain your exceptional situation in the text box.<br><br>Do the authors confirm that all data underlying the findings described in their manuscript are fully available without restriction?	
**Describe where the data may be found in full sentences. If you are copying our sample text, replace any instances of XXX with the appropriate details.**<br><br>• If the data are **held or will be held in a public repository**, include URLs, accession numbers or DOIs. If this information will only be available after acceptance, indicate this by ticking the box below. For example: *All XXX files are available from the XXX database (accession number(s) XXX, XXX.).*<br>• If the data are all contained **within the manuscript and/or Supporting Information files**, enter the following: *All relevant data are within the manuscript and its Supporting Information files.*<br>• If neither of these applies but you are able to provide **details of access elsewhere**, with or without limitations, please do so. For example:<br><br>*Data cannot be shared publicly because of [XXX]. Data are available from the XXX Institutional Data Access / Ethics Committee (contact via XXX) for researchers who meet the criteria for access to confidential data.*<br><br>*The data underlying the results presented in the study are available from (include the name of the third party*	All relevant data are within the manuscript and its Supporting Information files.

and contact information or URL).<br>• This text is appropriate if the data are owned by a third party and authors do not have permission to share the data.<br><br>* typeset	
Additional data availability information:	

Powered by Editorial Manager® and ProduXion Manager® from Aries Systems Corporation

Manuscript

Click here to access/download;Manuscript;PLOS ONE submission tSCS systematic review .docx

Title Page

Full Title:

Transcutaneous spinal cord stimulation and the generation of motor responses in individuals with spinal cord injury: a methodological review

Short Title:

Transcutaneous spinal cord stimulation and motor responses, a methodological review.

AUTHORS:

Clare Taylor[†1]*, Conor McHugh[†1], David Mockler[‡2], Conor Minogue[‡1], Richard B. Reilly[‡3,4,5] and Neil Fleming [‡1]

[1]*Department of Anatomy, School of Medicine, Trinity College, The University of Dublin, Ireland.*
[2]*John Stearne Medical Library, Trinity Centre for Health Sciences, School of Medicine, St. James's Hospital, Dublin 8, Ireland.*
[3] *Trinity Centre for Biomedical Engineering, Trinity College, The University of Dublin, Ireland.*
[4]*School of Engineering, Trinity College, The University of Dublin, Ireland.*
[5]*School of Medicine, Trinity College, The University of Dublin, Ireland.*

† Denotes graduate student author, ‡ Denotes professional author

*** Corresponding author**

Email: taylorc1@tcd.ie

Abstract

Background: Transcutaneous spinal cord stimulation (tSCS) is a non-invasive modality in which electrodes may stimulate spinal circuitries to produce a motor response. This review aimed to evaluate the methodology of studies using tSCS to generate motor activity in persons with spinal cord injury (SCI) and to appraise the quality of included trials.

Methods: A systematic search for studies published until June 2020 was made of the following databases: EMBASE, Medline (Ovid) and Web of Science. Two reviewers independently screened the studies, extracted the data, and evaluated the quality of included trials. The electrical characteristics of stimulation were summarised to allow for comparison across studies. In addition, the electrophysiological recording methods were evaluated.

Results: A total of 2222 articles were initially screened, of which 22 met the criteria for inclusion. Studies were divided into those using tSCS for neurophysiological investigations of reflex responses (n = 8) and therapeutic investigations of motor recovery (n = 14). The overall quality of evidence was deemed to be poor-to-fair (9.7 ± 5) based on the Downs and Black Quality Checklist criteria. The methods employed by included studies relating to stimulation parameters and outcome measurement varied extensively, although some trends are emerging in relation to electrode configuration and electromyographic (EMG) outcomes.

Conclusion: This review outlines the parameters currently employed for tSCS of the cervicothoracic and thoracolumbar regions to produce motor responses. However, to establish standardised procedures for neurophysiological assessments and therapeutic investigations of tSCS, further high-quality investigations are required, ideally utilizing consistent electrophysiological recording methods, and reporting common characteristics of the electrical stimulation administered.

Introduction

Transcutaneous spinal cord stimulation (tSCS) is a non-invasive form of neuromodulation in which electrodes are placed on the skin and used to stimulate the spinal circuitries via an electrical current (1-3). It has been proposed that this tool could provide us with a greater understanding of spinal functioning and enhance the rehabilitation potential for people with neurological disorders, such as spinal cord injury (SCI) (2, 4-6). As this is a novel modality under the relatively early stages of investigation, there is still much to learn about its implementation and clinical potential.

Modelling studies have demonstrated that electrical pulses delivered from spinal cord stimulation (SCS) preferentially depolarize sensory afferents in the posterior roots, which can elicit a motor reflex response (7, 8). This response has been termed a posterior root-muscle reflex [PRM (9)], multisegmental monosynaptic response [MMR, (5)], or transpinal evoked potential [TEP, (10, 11)], among other nomenclature. As an alternative to the H-Reflex, the study of the PRM reflex allows us to expand the neurophysiological assessment of sensory-motor transmission of stimuli and provides greater insights into the functioning of spinal circuitries across a multiple motor pools (4, 12).

Spinal stimulation via transcutaneous input is believed to be distinguished from direct stimulation of motor efferents, such as in traditional nerve or muscle stimulation techniques, due to the transsynaptic transmission of motor responses via monosynaptic or oligosynaptic pathways (13). Several studies have investigated the reflex nature of responses, using paired pulses to demonstrate post-activation depression (PAD), in which the amplitude of the second pulse of a pair is attenuated with respect to the first (14-22). Additionally, the inhibition of tSCS evoked responses via tendon vibration is consistent with the stimulation of reflex responses from Ia afferents (5, 22). Other studies have focused on alternative methods to demonstrate spinal neuromodulation of motor responses through outcomes such as increased

response latencies (2, 23), differential muscle activation patterns (24), phase-dependent modulation of reflex responses (5, 14) and the alteration of amplitudes subsequent to afferent input (25) or interlimb conditioning (26).

It is also theorized that SCS can modulate interneuronal spinal excitability and that this may account for the observed motor recovery when used in individuals with SCI (19, 27, 28). By activating networks such as central pattern generators (CPGs) and the propriospinal system (PSS), spinal excitability may be augmented and the threshold for motor impulse propagation lowered (29, 30). A CPG is a spinal network of neurons believed to be capable of generating a co-ordinated rhythmic motor output such as locomotion in the absence of input from supraspinal centres and/or afferent feedback (31). The PSS has been described as an interface between spinal segments that contributes to movement and rhythmic coordination (32, 33), as well as providing a background of subthreshold excitation (29, 34). The modulation of spinal networks and altered threshold for impulse propagation may explain the results of several studies using tonic spinal stimulation that have reported improved motor outcomes in chronically paralysed individuals (35-37), including the elicitation of voluntary motor responses to auditory commands (27, 38).

In the case of SCI, spinal neuromodulation may provide greater functional recovery beyond the capacity of currently available therapies, particularly after more severe or chronic injury (29, 39). Thus far, a selection of studies investigating the effects of tSCS on motor rehabilitation in chronic SCI have published cases of improved lower limb (19, 40-42), trunk (36) and upper limb functioning (35, 43, 44). Stimulation therapy may be a promising means to improve motor capacity, particularly when combined with other complementary interventions to provide synergistic rehabilitation, such as partial weight-bearing therapy and treadmill training (19, 25, 45) and/or pharmacological agents (38, 41, 44). Despite these promising initial results, a recent review evaluating the therapeutic effects of tSCS on motor

recovery in individuals with SCI reported that due to small heterogenous sample sizes and the low methodological quality of reviewed studies no conclusions can be drawn on its effectiveness (46).

Presently, there is also a lack of consensus surrounding optimal stimulation parameters and experimental protocols. Therefore, the extent of this methodological variability would benefit from a systematic evaluation in order to synthesize the information on currently employed parameters and provide recommendations to enhance the development of future studies investigating the properties and efficacy of tSCS. As such, the objective of this systematic review was to methodologically appraise studies which used tSCS to generate motor activity in persons with SCI. In doing so, this review sought to critique the quality of included trials, review intervention parameters employed and compare the methods of evaluating motor responses.

Methods

A systematic review of the literature was undertaken using the methodology described by the Preferred Reporting Items for Systematic Review and Meta-Analysis (PRISMA) Protocols Statement (47).

Search strategy

An extensive literature search was carried out using the following electronic databases: EMBASE, Medline (Ovid) and Web of Science. It included studies from a 15-year period, from 1995 to June 2020. The initial search was kept broad to in an attempt to capture all possible spinal stimulation studies using varying nomenclature. The search was built with the help of a research librarian (DM) based on anchoring terms from the following categories: ***spinal cord stimulation, spinal cord injury and motor response generation***. Search terms were expanded using a vast list of alternative terminologies, truncations, and abbreviations. The exact search

algorithm and medical subject heading (MeSH) terms used with each engine are presented in S1 Appendix. Additional relevant publications were also sought out by retrospectively completing a manual search of the bibliographies of all included studies and by manually searching for other publications from authors of tSCS studies that were identified in the search.

Study selection procedure

Two independent reviewers (CT, CMcH) completed an initial title screen to remove any highly irrelevant papers. The eligibility criteria (Table 1) were designed based on the PICO model (Population, Intervention, Comparison, Outcome). Pilot testing of the exclusion criteria was conducted using a subset of 150 abstracts screened by both reviewers and the reasons for exclusion were documented. The reviewers then completed the abstract screening and a Cohen's Kappa of 0.88 was reached. This correlation was deemed sufficient. Finally, the full texts were reviewed for inclusion and all reasons for exclusion were recorded. If there was any uncertainty about inclusion, a third reviewer (NF) was consulted until a consensus was reached. The independent reviewers were not blinded to the study authors, institutes, or journal titles. As there were a small number of publications meeting the inclusion criteria, we did not require a minimum sample size. The literature search was last performed on the 30th of June 2020.

	INCLUSION	EXCLUSION
**PARTICIPANTS**	- Aged > 18 years<br>- A primary diagnosis of spinal cord injury (any level, complete or incomplete).	- Animal studies<br>- Participants < 18 years of age
**INTERVENTION**	- Transcutaneous spinal cord stimulation aimed at producing a motor response.<br>- Pulsed and continuous electrical spinal stimulation protocols.	- Magnetic stimulation or direct current stimulation<br>- Peripheral stimulation such as Functional Electrical Stimulation (FES) or Neuromuscular Electrical Stimulation (NMES)<br>- Paired Associative Stimulation (PAS)<br>- Epidural spinal cord stimulation (eSCS).
**COMPARISON**	- No intervention, sham intervention, or pre-post analysis	
**OUTCOMES**	- A measure of motor activity in a targeted muscle/muscle groups by EMG recordings	- The primary outcome selected and reported on measures pain, autonomic function, or spasticity
**DATA ANALYSIS**	- Study must report details pertaining to the transcutaneous spinal cord stimulation parameters utilised	- Studies that fail to specify any stimulation parameters
**PUBLICATION TYPE**	- Original primary data from a prospective interventional, quasi-experimental, or observational study	- Review articles, conference proceedings, expert opinions, or any other secondary publication
	- Published in peer reviewed journal over last 15 years from 1995 to June 2020.	- Published <1995
	- Published in English	- Abstract or full text not available in English

Table 1. The eligibility criteria to determine suitable studies for inclusion in the full-text systematic review.

Quality appraisal

In order to appraise the quality of the included full texts, the Downs and Black (D&B) Checklist was employed (48). This tool has been used to evaluate non-randomised controlled trials (RCTs) in other systematic reviews pertaining to populations with SCI (49-51) and its use is recommended by the SCIRE (Spinal Cord Injury Research Evidence) Research Team (52). The D&B Checklist has also been recommended for use in assessing non-RCTs due to its psychometric properties (53, 54).

Two independent reviewers (CT, CMcH) conducted the quality appraisal and any disagreement was discussed with a third reviewer (NF) until consensus was reached. The D&B Checklist is a 27-item list that evaluates methodological strengths and weaknesses of articles based on the categories of *(1) Reporting, (2) Internal Validity (Bias), (3) Internal Validity (Confounding), (4) External Validity* and *(5) Power* (48). Power level calculations (1-β error probability) for the checklist were made using the G*Power Application (55) and analysis was derived from the statistical tests applied to the main study findings. The following marks were awarded: 1 point for a power level of 70%, 2 points for power level of 80%, 3 points for power level of 85%, 4 points for power level of 90%, 5 points for power level of 95%. The modified version of the D&B Checklist was not used, as the authors felt it important to adequately represent the sufficient powering of studies as per the original. The following rounded cut-off points were used to categorize studies by quality (56): excellent (91%–100%), good (71%–90%), fair (51%–70%), and poor (0%–50%).

Data Extraction

Results were generated from data extracted to standardised spreadsheets which included (i) study type, (ii) sample characteristics and clinical variables, (iii) intervention parameters, (iv) outcome measurements (v) Electromyography (EMG) data collection and signal processing (vi) and safety/adverse events. Table results were pooled by two study members until

consensus was reached, and disagreements were discussed with the third reviewer. Studies investigating similar objectives were grouped together for comparison, in particular, a distinction was made between neurophysiological experiments and therapeutic investigations seeking motor rehabilitation.

The electrical and timing characteristics of the stimulation signals used in tSCS vary widely, making comparisons between studies difficult. Moreover, there is a lack of consistency in the definition of these parameters. This study sought to clearly define key stimulation parameters and descriptors and, where possible, extract data from each publication according to these definitions. *Figure 1.* shows typical waveforms for constant current pulsed stimulation and identifies selected characteristics, while Table 2 defines the parameters that were used to characterise the tSCS administered.

Figure 1. (submitted as separate file)

Table 2. Summary of stimulation parameters and how they are defined.

Parameter	Symbol	Unit	Description
Pulse Interval	T	ms	The time interval between pulses of a sequence
Pulse Frequency	f	Hz	The inverse of the pulse interval, f=1/T, is the number of pulses per second
Phase duration	t_1	ms	The duration of the leading phase
Pulse Amplitude	i	mA	Current amplitude measured baseline to peak
Phase Charge	q_c	µC	Total charge in the phase
Pulse duration	p	ms	The sum of $t_1+t_2+t_3$
Carrier Frequency	f_c	Hz	Frequency of a carrier waveform which is modulated by the stimulation waveform
Carrier-on-time	tc_1	µs	Phase duration of carrier waveform
Carrier Period	Tc	µs	Inverse of carrier frequency
Phase charge density	q_d	µC/cm^2	The phase charge per unit electrode area

Root mean square current	i_{rms}	mA	$i_{rms} = \sqrt{\frac{1}{T}\int_0^T i(t)^2\,dt}$
Electrode current density	j_e	mA/ cm^2	$j_e = i_{rms}/A$
Electrode Area (active)	A	cm^2	The area of electrical contact at the skin. (assumed uniform current distribution within electrode)

Table 2. Summary of stimulation parameters and a detailed description of how they are defined

The root mean square (RMS) current is useful for estimating average electrical power and therefore the heat generating capacity of a waveform, $P_{avg} = i_{rms}^2 R$. For a square wave such, as at *Figure 1b*, the RMS current calculation simplifies to:

$$i_{rms} = i\sqrt{\frac{t1}{T}}$$

Or, for a typical symmetric biphasic waveform like that at *Figure 1a*, the calculation would be:

$$i_{rms} = i\sqrt{\frac{t1 + t2}{T}}$$

For the descriptions of other details of included studies, ranges are given with the mean ± standard deviation. Due to the heterogeneity in the methods used to evaluate the outcomes and the diverse experimental methodologies, a meta-analysis was not possible, and a descriptive qualitative review was conducted.

Results

Literature search and selection

Of the 3435 articles identified (Embase: 1739, Medline (Ovid): 1355, Web of Science: 341), 2222 were taken to title and abstract screening after the duplicates were removed. After the removal of 2136 articles from title and abstract screening, 86 full texts were evaluated for eligibility. Finally, 22 articles that assessed the ability of tSCS to directly generate motor responses in individuals with SCI were included in this review (Figure 2).

Figure 2. PRISMA Flow Diagram of screening and selection processes. (submitted as separate file)

Study characteristics

Studies were categorized as neurophysiological assessments if their objective was to investigate the properties, mechanisms or effects of tSCS on outcomes related to nervous system functioning (n=8), whereas studies were labelled as therapeutic if they aimed to enhance motor rehabilitation and recovery in patients with SCI (n=14). In therapeutic investigations, tSCS was commonly combined with simultaneous rehabilitative interventions such as physical therapy, treadmill training, body weight support and the use of exoskeletons or pharmacological agents (Table 3). Of the 22 included studies, 7 were case reports, 5 were case series, 3 were crossover trials, 6 were quasi-experimental studies (non-equivalent control group or nonrandomised intervention design) and one was a non-randomised control trial.

Participant demographics

A total of 153 participants with SCI were recruited across the 22 studies to receive tSCS and their characteristics are described in Table 3. Further analysis only includes data from participants with SCI due to the purposes of this review. The sample sizes in included studies were generally modest (n = 7 ± 6). Neurophysiological investigations tended to have larger samples (n = 10 ± 5) than therapeutic investigations (n = 6 ± 6). A large range of ages, from 18 to 70 (mean 35 ± 13 years), injury classifications, from the level of C2 - L2, and impairment

levels, AIS A - D classifications, were represented across the included studies. Studies explored the effects of tSCS on different injury chronicity's, from one year to 43 years post-injury occurrence, however, no published studies investigated the use of tSCS at < 1-year post injury.

Table 3. Study characteristics and participant demographics

Study	Design	Simultaneous Interventions	Other Subjects (*n*)	Participants with SCI Demographics					
				Sample (*n*)	Age (years) Mean (SD)	Gender	AIS	Level	Time Since Injury (years) Mean (SD)
Dy *et al.*, 2010 (14)	NP, QE	Treadmill and BWS	9 NI	9	32.6 (9.2)	M=9	A	C5-T7	6.4 (9.4)
Hofstoetter *et al.*, 2013 (6)	TP, CR	Treadmill	-	1	29	F=1	D	T9	11
Gad *et al.*, 2015 (57)	TP, CR	Exoskeleton	-	1	38	M=1	A	T9	4
Gerasimenko *et al.*, 2015 (38)	TP, CS	Assisted movement, Buspirone	-	5	31.4 (16.8)	M=5	B	C5-T4	3.2 (1.6)
Hofstoetter *et al.*, 2015 (18)	TP, CS	Treadmill	-	3	32.7 (5)	M=2, F=1	D	C5, T9	10.7(1.5)
Bedi, 2016 (58)	TP, CR	NIL	-	1	25	M=1	C	T12	-
Emeliannikov *et al.*, 2016 (15)	NP, CS	Seated gait device, pharmacology	-	10	39.1 (11.3)	M=7, F=3	A-D	T5-L2	4.8 (4.2)
Minassian *et al*, 2016 (19)	TP, CS	RDGO, Treadmill and BWS	-	4	39.5 (17.1)	M=3, F=1	A	C8-T8	2.8 (1.4)
Gad *et al.*, 2017 (41)	TP, CR	Exoskeleton, Buspirone	-	1	40	M=1	A	T9	4
Freyvert *et al.*, 2018 (44)	TP, CrT	Buspirone, hand grip exercises	-	6	19.2 (1.3)	M=4, F=2	B	C5-C8	2.4 (0.9)
Gad *et al.*, 2018 (43)	TP, CS	Hand grip exercises	-	6	40.2 (16.6)	M=5, F=1	B, C	C4-C8	8.0 (7.7)
Hofstoetter *et al.*, 2018 (16)	NP, QE	PT	7 SCI eSCS	10	39.7 (20.1)	M= 7, F=3	A, C, D	C4-T7	4.5 (2.8)
Inanici *et al.*, 2018 (35)	TP, CR	Activity-based PT	-	1	62	M	D	C3	2
Rath *et al.*, 2018 (36)	TP, RCrT	NIL	-	8	29.4 (7.7)	M=7, F=1	A, C	C4-T9	7.3 (3.3)
Hofstoetter *et al.*, 2019 (17)	NP, QE	NIL	10 NI	10	40.1 (18)	M=8, F=2	A, C, D	C4-T7	9.7 (12.5)
Murray and Knikou, 2019 (20)	NP, QE	NIL	10 NI	10	36.3 (11.2)	M=7, F=3	A-D	C6-T12	8.8 (8.1)
Sayenko *et al.*, 2019 (42)	TP, RCrT	Stand Training	-	15	31.2 (8.7)	M=12, F=3	A-C	C4-T12	6 (3.2)
Alam *et al.*, 2020 (40)	TP, CR	Stand Training and Treadmill	-	1	48	F=1	D	C7	21
Atkinson *et al.*, 2020 (26)	NP, QE	NIL	15 NI	18	29 (7)	M=16, F=2	A-D	C2-T6	4.6 (3.1)
Militskova *et al.*, 2020 (25)	NP, CR	PT, treadmill and BWS	-	1	21	F=1	A	T11	1
Shapkova *et al.*, 2020 (37)	TP, NRCT	Exoskeleton	16 SCI Control	19	31.2 (8.6)	M=15, F=4	A-C	C8-L2	4.6 (3.3)
Wu *et al.*, 2020 (23)	NP, QE	NIL	14 NI, 4 ALS	13	45.9 (13.7)	M=10, F=3	B-D	C2-C8	10.8 (5.9)

Table 3: Details of study characteristics and the demographics of included participants. Abbreviations: AIS; ASIA impairment scale, ALS; amyotrophic lateral sclerosis, BWS; Body weight support, CrT; crossover trial, eSCS; epidural spinal cord stimulation, F; female, M; male,

NP; neurophysiological investigation, NRCT; non-randomised controlled trial, PT; physical therapy, QE; quasi-experimental study, RDGO; Robotic driven gait orthosis, RCrT; randomised crossover trial, SCI; spinal cord injury, SD; standard deviation, TP; therapeutic investigation

Quality appraisal

The quality of included trials was evaluated using the D&B Checklist (48) and this tool deemed the overall evidence quality to be poor-to-fair, with results ranging from 3 to 19, out of a possible score of 32 (Tables 4 and 5). The mean score across all trials was 9.7 ± 5, with 11.1 ± 5.4 for neurophysiological and 8.9 ± 4.9 for therapeutic investigations. In particular, low scores were repeatedly awarded for external validity and selection bias, and the majority of studies were deemed insufficiently powered.

Table 4. **Quality of Evidence Assessment – Neurophysiological Investigations**

Study	Reporting	External Validity	Internal Validity (Bias)	Internal Validity (Selection Bias)	Power	Quality Score	Percentage	Evidence Category
Dy *et al.*, 2010 (14)	6	0	4	0	0	10	31%	Poor
Emeliannikov *et al.*, 2016 (15)	2	0	2	0	0	4	13%	Poor
Hofstoetter *et al.*, 2018 (16)	7	0	4	1	0	12	38%	Poor
Hofstoetter *et al.*, 2019 (17)	8	0	5	1	5	19	59%	Fair
Murray and Knikou, 2019 (20)	7	0	3	0	1	11	34%	Poor
Atkinson *et al.*, 2020 (26)	6	0	4	0	0	10	31%	Poor
Militskova *et al.*, 2020 (25)	4	0	1	0	0	5	16%	Poor
Wu *et al.*, 2020 (23)	9	0	3	1	5	18	56%	Fair
	**/11**	**/3**	**/7**	**/6**	**/5**	**/32**	**/100%**	

Table 4. *Results from the quality appraisal of neurophysiological investigations using the Down's and Black Checklist.*

Table 5. **Quality of Evidence Assessment– Therapeutic Investigations**

Study	Reporting	External Validity	Internal Validity (Bias)	Internal Validity (Selection Bias)	Power	Quality Score	Percentage	Evidence Category
Hofstoetter *et al.*, 2013 (6)	1	0	2	0	0	3	9%	Poor
Gad *et al.*, 2015 (57)	1	0	2	0	0	3	9%	Poor
Gerasimenko *et al.*, 2015 (38)	4	0	2	0	0	6	19%	Poor
Hofstoetter *et al.*, 2015 (18)	2	0	2	0	0	4	13%	Poor
Bedi, 2016 (58)	4	0	3	0	0	7	22%	Poor
Minassian *et al.*, 2016 (19)	5	0	4	0	0	9	28%	Poor
Gad *et al.*, 2017 (41)	3	0	0	0	0	3	9%	Poor
Freyvert *et al.*, 2018 (44)	7	0	5	2	0	14	44%	Poor
Gad *et al.*, 2018 (43)	9	0	2	0	0	11	34%	Poor
Inanici *et al.*, 2018 (35)	5	0	3	1	0	9	28%	Poor
Rath *et al.*, 2018 (36)	7	0	4	1	0	12	38%	Poor
Sayenko *et al.*, 2019 (42)	6	0	3	4	4	17	53%	Fair
Alam *et al.*, 2020 (40)	3	0	2	4	0	9	28%	Poor
Shapkova *et al.*, 2020 (37)	8	1	5	0	3	17	53%	Fair
Total Maximum Score	**/11**	**/3**	**/7**	**/6**	**/5**	**/32**	**/100%**	

Table 5. *Results from the quality appraisal of therapeutic investigations using the Down's and Black Checklist.*

Methodological Evaluation

The methodologies of selected studies were reviewed to outline the common procedures for stimulation implementation and outcome evaluation. The stimulation variables selected determine the electrical field generated and subsequent motor responses, and the outcomes used to evaluate these responses are essential in understanding the utility of the parameters selected and the overall effectiveness of tSCS. Factors such as safety and adverse events are also critical to a methodological review.

Electrode configurations

Details of stimulation parameters and electrical characteristics are outlined in Tables 6 and 7. The electrode configurations with regard to position and location varied substantially across experiments. The cathode was positioned dorsally over the vertebral column in the majority of studies (n = 13), or electrodes were used that alternated polarity within a biphasic pulse (n = 5). Studies placed the stimulating electrode/s either at the midline over the vertebrae (14, 17, 20, 25, 26, 38, 41-43), or paravertebrally (6, 16, 18, 19, 58). The majority of studies targeted a single site, however, 8 out of 14 therapeutic investigations favoured the stimulation of multiple sites simultaneously (35, 36, 38, 40-43, 57).

The most common vertebral level stimulated for targeting lower limb motor activity was T11-T12 (n=17) and/or L1-L2 (n=6). Two studies placed electrodes within the range of T9-L2, but adjusted the exact positions based on motor responses (25, 26). An additional secondary stimulating electrode was also placed on the coccygeal bone during three experiments (38, 41, 57). For the upper limb responses, the cathode site varied substantially across the four studies and was placed on C5 (44), T2-T4 (23), or C3-C4 simultaneously with C6-C7 (35, 43).

The anode location selected for experiments targeting lower limb motor responses varied between the anterior superior iliac spine (ASIS) and iliac crests (n=6) or para-umbilically over the anterior abdomen (n=7), with one study recording the use of both locations depending on

patient comfort (20). In studies of upper limb responses, the iliac crests or ASIS were chosen by three investigations (35, 43, 44) with only Wu, Levine (23) placing the anode on the anterior neck.

Table 6. Stimulation parameters selected by studies carrying out neurophysiological assessments into the properties of spinal cord stimulation with SCI participants

		ELECTRODES			STIMULATION PROTOCOL			ELECTRICAL CHARACTERISTICS	
**Study**	**Patient position**	**Size/shape [Area]**	**Polarity**	**Location**	**Description**	**Frequency**	**Intensity**	**Phase charge (µC)**	**Phase Charge Density (µC/cm²)**
Lower limb responses									
Dy *et al.*, 2010 (14)	Lying prone, BWS standing, BWS stepping	ø 2.5 cm [4.9 cm²]<br>Pair 5.0 x 10.2 cm	Cathode<br>Anodes	T11-T12<br>Iliac crests	Single, t_1= 1ms, monophasic square wave pulses	1) Prone/ Standing: 0.5 Hz<br>2) Stepping: 0.25-0.33 Hz	24.7 - 83mA<br>Set to where consistent responses observed in all measured muscles in standing	30 – 83	16.9
Emeliannikov *et al.*, 2016 (15)	Seated	-<br>-	-<br>-	T11-T12<br>-	t_1= 1ms paired pulses (50ms inter-pulse interval)	0.3 Hz for H-Reflex<br>NS for MMR	30 – 80 mA<br>Lowest amplitude to completely supress the second stimulus of a pair	30 - 80	Not available
Hofstoetter *et al.*, 2018 (16)	Lying supine	Pair ø 5 cm [2 x 19.6 cm²]<br>8 x 13 cm	Alternating (anode first pulse, cathode second)<br>Alternating	T11-T12 paravertebrally<br>Para-umbilically lower abdomen	Charge balanced, symmetric biphasic rectangular t_1= 1ms	-	32-86 mA Adjusted to reach target threshold >100uV in all muscle groups studied	32 – 86	2.2
Hofstoetter *et al.*, 2019 (17)	Lying supine	5 x 9 cm [45 cm²]<br>Pair 8 x 13 cm	Alternating (anode first pulse, cathode second)<br>Alternating	T11 - T12<br>lower abdomen	charge balanced, symmetric biphasic rectangular t_1= 1ms	-	Adjusted to elicit control-PRM reflexes in the right soleus with amplitudes that best matched the control-H reflexes and to elicit PRM reflexes in other muscles studied	Not available	Not available

Murray and Knikou, 2019 (20)	Lying supine	10.2 x 5.1 cm [52 cm^2]	Cathode	T10 - L1/L2	1) Intervention: alternating suprathreshold and subthreshold stimulation (60 mins/session), monophasic square wave t_1= 1ms	1) 0.2Hz	Selected based on threshold to produce right soleus evoked potential (96.9 ± 24 mA). Treatment sessions ranged from 0.4 - 4.3 times this resting threshold	97	1.9
		Connected pair 10.2 x 5.1 cm²	Anode	Para-umbilically/ iliac crests	2) Assessment: monophasic square wave t_1= 1ms	2) 0.1, 0.125, 0.2, 0.33, 1.0 Hz	From below motor threshold until plateau reached	417	8.0
Atkinson *et al.*, 2020 (26)	Lying supine	ø 1.8 cm [2.6 cm^2]	Cathode	midline T9-T10 (n=1), T10-T11(n=7), T11-T12 (n=6), T12-L1 (n=1)	Single, monophasic square wave pulses, t_1= 1ms	-	0-100mA or until response magnitude plateaued	0 to 100	39.3
		Pair 5 x 9 cm	Anode	anterior superior iliac spines					
Militskova *et al.*, 2020 (25)	Lying supine, BWS standing	ø 2.5 cm [4.9 cm^2]	Stimulating	midline T9-T10, T10-T11, T11-T12, T12-L1, and L1-L2	monophasic rectangular pulses t_1= 1ms	-	30-100mA or maximum tolerated	30 to 100	20.4
		Pair 4 x 2 cm	Reference	lower abdomen					
Upper limb responses									
Wu *et al.*, 2020 (23)	Seated	Pair 5 x 10cm [2 x 50 cm^2]	1) Alternating polarity 2) Cathode posterior for majority	4cm caudal to C7 (T2-T4)	1) anode posterior t_1= 1ms biphasic, 2) cathode posterior t_1= 1ms biphasic, 3) cathode posterior t_1= 0.5ms biphasic, 4) cathode posterior t_1= 1ms monophasic	0.2Hz	80 - 175% of RMT, (RMT = 5.5 - 51 mA)	89 µC mono<br>89 µC biph	1.8<br>1.8
		Pair 5 x 10cm	1) Alternating polarity 2) Anode anterior for majority	1-2cm above sternal notch (C4-C5 levels anteriorly)			threshold calculated as enough to elicit > 50uV in 5/10 reps		

Table 6: *Parameters selected by neurophysiological assessments investigating the effects of tSCS on spinal cord functioning in individuals with SCI.* ***Abbreviations:*** *PRM; posterior root-muscle, RMT; resting motor threshold. Where more than one test protocol existed within a given publication, the protocols were detailed using numerical listing: 1) 2) 3) etc.*

Table 7. Stimulation parameters selected by therapeutic studies investigating the effects of transcutaneous spinal cord stimulation for motor recovery

		ELECTRODES			STIMULATION PROTOCOL				ELECTRICAL CHARACTERISTICS		
**Study**	**Position/ Action**	**Size/shape [Area]**	**Polarity**	**Location**	**Description**	**Frequency**	**Intensity**	**Duration**	**Pulse Charge (μC)**	**Current RMS (mA)**	**Current Density (mA/cm^2)**
Hofstoetter *et al.*, 2013 (6)	Upright/stepping on treadmill	Pair ø 5cm [2 x 19.6 cm^2]<br><br>Pair 8 x 13cm	-<br><br>-	Sgl: T11/T12<br><br>Lower anterior abdomen	Sub-motor threshold, charge balanced, symmetric, biphasic rectangular pulses of t_1= 1ms	30Hz	18V	-	Not available	Not available	Not available
Gad *et al.*, 2015 (57)	Standing, supine /exoskeleton stepping, voluntary movement	-	-	Mult: T11 and Co1	Tonic	T11: 30Hz Co1: 5Hz	-	3 x 20 mins (T11, Co1, both)	Not available	Not available	Not available
Gerasimenko *et al.*, 2015 (38)	Side-lying/ gravity-neutral stepping	ø 2.5cm [4.9 cm^2]<br><br>Pair 5 x 10.2cm^2	Cathode<br><br>Anode	Mult: T11-T12 and coccyx 1 (Co1)<br><br>Iliac crests	Monopolar rectangular stimuli, t_1= 1ms	T11: 30Hz (+10kHz *cf*) Co1: 5Hz (+10kHz *cf*)	80-180mA, stepping motor threshold	3 x 3 mins (T11, Co1, both)	40-90<br><br>40-90	9.8-22<br><br>4.0-9.0	2.0-4.5<br><br>0.8-1.8
Hofstoetter *et al.*, 2015 (18)	Standing/ treadmill stepping	Pair ø 5cm [2 x 19.5 cm^2]<br><br>Pair 8 x 13cm	Alternating (anode 1st pulse phase, cathode 2nd)<br><br>Alternating	Sgl: T11/T12 paravertebrally<br><br>Para-umbilically	Charge-balanced, symmetric, biphasic rectangular t_1= 1ms	30Hz	18-27 V, 86% of reflex threshold (P1), 71% (P2), 80% (P3).	-	Not available	Not available	Not available
Bedi, 2016 (58)	Voluntary and passive movement	Pair 4.5 x 9 cm [2 x 41 cm^2]	-	Sgl: T10-L1 paravertebrally	-	30, 50, 70, 90 Hz (+2.5 kHz *cf*)	Raised to sensory threshold	45 mins per frequency			

Minassian *et al.*, 2016 (19)	Supine, Standing/ assisted treadmill stepping	Pair ø 5 cm [2 x 19.6 cm^2]	Cathode	Sgl: T11- T12, paravertebrally, 1cm apart	Rectangular monophasic t_1= 1ms	30Hz	P1: 140mA<br>P2: 100mA<br>P3:170mA<br>P4: 125mA	10 gait cycles	P1: 140<br>P2: 100<br>P3: 170<br>P4: 125	24.25<br>17.32<br>29.44<br>21.65	0.62<br>0.44<br>0.75<br>0.55
		Pair 8 x 13cm	Anode	Abdomen			Increments of 5mA until reflex threshold				
Gad *et al.*, 2017 (41)	Standing, supine/ exoskeleton stepping, voluntary movement	ø 2.5cm [4.9 cm^2]	Cathode	Mult: T11-T12 and coccyx 1 (Co1)	-	T11: 30Hz<br>Co1: 5Hz<br>Tc=100µs *cf*	-	3 x 20 mins/ session	Not available	Not available	Not available
		5 x 10.2cm^2	Anode	Iliac crests							
Sayenko *et al.*, 2019 (42)	Standing/ standing balance exercises, sit-to-stand	ø 3.2cm diameter [8 cm^2]	Cathode	Mult + sgl:<br>1) T11 and/or L1<br>2) L1	Monophasic t_1= 1ms pulses	1) T11/L1: 5, 15, 25 Hz Tc=100µs *cf*	1) T11/L1: Up to 150mA	-	75	16.7 (@ 25 Hz)	2.1
		Pair 7.5 x 13 cm	Anode	iliac crests		2) L1: 15Hz	2) L1: Up to 100mA		75	8.7 (@15 Hz)	1.1
Alam *et al.*, 2020 (40)	Standing, sitting, supine/ standing, stepping and voluntary movement training	Pair ø 3.2cm [2 x 8 cm^2]	Alternating	Mult: T11 and L1	1) biphasic with tc_1=50µsec t_1= 100 µs	1) 20 Hz /100 µs	1) T11: 105mA, L1: 100mA	3 x ~10 mins and 3 x 2-3 mins/session	5.3	3.8	0.3
		Pair 6x9 cm		iliac crests	2) tonic biphasic stimulation with tc_1=50µs t_1= 100 µs	2) 30Hz /100 µs	2) T11: 95mA, L1: 90mA		4.8	4.7	0.3
					3) tonic biphasic stimulation with tc_1=50µs t_1= 1ms	3) 20-30Hz/1ms	3) T11: 20-120mA, L1: 20-120mA		60	14.7 (30Hz)	0.9
Shapkova *et al.*, 2020 (37)	supine, standing/ exoskeleton walk training	Pair 3 x 4 cm [2 x 12 cm^2]	Cathode	Sgl: T12 vertebrae	t_1= 0.5ms monophasic square wave	G1: 1 Hz, G2: 3 Hz, G3: 67 Hz	1.3-1.4 x motor threshold	~41-53 mins/session	50	G1: 2.2<br>G2: 3.9<br>G3: 18.3	0.1<br>0.2<br>0.8
		Pair 3 x 4 cm	Anode	central abdomen			5-100mA				

Upper limb responses											
Freyvert *et al.*, 2018 (44)	Voluntary hand contractions	-	Cathode<br><br>Grounding	Sgl: C5<br><br>ASIS		5-30Hz for 15-30 mins	20-100mA	15-30 mins/session	Not available	Not available	Not available
Gad *et al.*, 2018 (43)	Voluntary hand contractions	ø 2cm diameter [3.1 cm^2]<br><br>Pair 5.0 x 10cm^2, rectangular	Cathode<br><br>Anode	Mult: C3-C4 and C6-C7<br><br>Iliac crests	1) Pulsed monophasic t_1=ms<br><br>2) Continuous biphasic or monophasic t_1=ms	1) 1Hz<br><br>2) 30 Hz + (Tc=100µs *cf*)	1) 10-200mA<br><br>2) 70-210mA	-	100	4.5 (1 Hz)<br><br>25.7 (@30Hz, bi)<br><br>36.4 (@30Hz mono)	1.4<br><br>8.2<br><br>11.6
Inanici *et al.*, 2018 (35)	Upper limb activity-based PT	Pair x ø 2.5cm [2 x 8 cm^2]<br><br>Pair x 5 x 10cm	Cathode<br><br>Anodes	Mult: midline C3-C4 and C6-C7<br><br>iliac crests	1) Continuous 60 ± 20 mins biphasic t_1=1 ms<br><br>2) Pulsed monophasic rectangular t_1= 1ms bursts	1) 30 Hz + (Tc=100µs *cf*)<br><br>2) 1Hz	1) 80-120mA<br><br>2) 10-120mA at 10 mA intervals	1) 60 ± 20 mins/session<br><br>2) pulsed	60<br><br>60	14.7 (30Hz)<br><br>2.7 (1Hz)	1.5<br><br>0.3
Trunk responses											
Rath *et al.*, 2018 (36)	Sitting/ seated balance tasks	2 x ø 3.2cm [2 x 8 cm^2]<br><br>Pair 7.5 x 13cm	Cathode<br><br>Anode	Mult: T11 and L1<br><br>Iliac crests	Monophasic, rectangular 1ms pulses	T11: 30Hz (Tc=100µs *cf*)<br>L1: 15Hz (Tc=100µs *cf*)	1) 10-150mA to detect motor threshold<br>2) constant sub-threshold T11 : 25-100mA<br>L1 : 5-80mA	3-4 x ~1-2 mins/session	50<br><br>40	12.25 (T11)<br><br>6.93 (L1)	1.52<br><br>0.86

Table 7: Parameters selected by therapeutic studies investigating the effects of tSCS on motor rehabilitation., Abbreviations: ASIS; Anterior Superior iliac Spine, cf; carrier frequency, Co1; coccyx 1, G; group, mult; multiple stimulation levels, P; participant, PT; physical therapy, sgl; single stimulation level.
Where more than one test protocol existed within a given publication, the protocols were detailed using numerical listing: 1) 2) 3) etc.

Electrical dosage

Clear differences in dosage arose between neurophysiological and therapeutic investigations. In neurophysiological investigations, tSCS was typically delivered using isolated single or paired pulses with long refractory periods allowing a return of resting membrane potential. Frequencies, when outlined, were therefore typically low ranging from 0.1 Hz (20) to 1 Hz. Delivered current in neurophysiological investigations ranged between 5.5mA to 120.9mA with only one study exceeding a maximum of 100mA (20). A variety of criteria were used to determine the stimulation intensities, for example, the point at which threshold responses were observed in some (20, 23) or all muscles (14, 16, 17), maximum tolerance (25), response magnitude plateau (26) or the lowest amplitude that completely supressed the second stimulus of a pair (15). The majority of neurophysiological experiments reported using a square or rectangular monophasic current waveform with 1ms pulse width, with just two studies using biphasic pulses (16, 17), and one which trialled both (23).

In contrast, therapeutic investigations typically reported the application of continuous pulse trains, with a burst frequency of 5 – 30 Hz and an intra-pulse carrier frequency of 2.5 – 10 kHz (35, 36, 38, 42, 43, 58). The use of this intra-pulse carrier frequency is poorly justified and appears to be for analgesic purposes, although no evaluation of this could be identified. Other therapeutic experiments selected simplified phase characteristics with either biphasic or monophasic rectangular-waves with a frequency ranging from 1 – 90 Hz, and 20-30 Hz the most commonly occurring selection (6, 18, 19, 37, 40, 44, 57). The duration of therapeutic stimulation varied from bouts of <5 mins (36, 38, 40) to > 45 mins (35, 37, 58) and was generally paired with concomitant rehabilitative activities. Recorded current ranges in therapeutic experiments were larger than neurophysiological investigations, reaching a maximum of 180mA in the thoracolumbar region (38) and 210mA in the cervical region (43). Intensity criteria was not always explicitly specified. Some studies note that it was based off

sufficient levels to reach desired muscle responses (38, 43), perceived sensory thresholds (58) or the amplitude at which reflex threshold was reached (19). The pulse width was between 0.5 to 1 ms per phase with rectangular waveforms and the majority applied monophasic pulses, with the exception of three studies that selected biphasic (6, 18, 40) and two studies that tested both (35, 43). Two studies used voltage pulses (6, 18) and the resulting current amplitude was not available.

Electrical Characteristics

The variances in electrode sizes and configurations along with differences in dosage parameters such as amplitude, frequency and pulse duration make it difficult to compare the electrical characteristics of stimulation across studies. We have therefore attempted to calculate common characteristics that were gleaned from the available data. The pulse charge was reasonably consistent in the neurophysiological investigations, in the range 30 to 100 μC, although one study did exceed this value (20). The resulting charge density at the spinal electrode varied enormously between studies because of the range of electrode sizes used, 0.03 to 7.9 $\mu C/cm^2$

The therapeutic investigations used sustained trains of pulses and the resulting current and current density was compared. Root-mean-square current was in the range 2.2 to 36.4 mA, with most studies below 20 mA. Once again, the variation in electrode area led to a wide range of current densities between studies, 0.1 to 4.5mA/cm^2, with one study exceeding this due to a high current combined with a small electrode area (43).

Participant positioning

The majority of neurophysiological investigations were conducted with subject in either supine (16, 17, 20, 26) or seated (15, 23) positions. Only one study was carried out with participants standing and prone (14). One investigation compared a number of varying positions to

investigate positional effects (25). In contrast, the majority of therapeutic interventions targeting lower limb responses were conducted in an upright standing position (6, 18, 19, 37, 40-42, 57) and/or while supine (19, 37, 40, 41, 57). Stimulation targeting trunk control and sitting balance was carried out in a seated position (36) and positional details were omitted in therapeutic studies investigating upper limb functioning.

The reflex nature of tSCS responses

Only two therapeutic investigations (18, 19) assessed the nature of motor responses generated by tSCS, while in contrast, all neurophysiological investigations recorded the reflex origin of evoked responses. Primarily the transynaptic modulation of responses was demonstrated using the paired pulse paradigm in which two pulses were delivered with a short conditioning-test interval (CTI) to demonstrate PAD of the second response. Interstimulus intervals between 30-50ms were generally selected to demonstrate PAD (15, 16, 23, 25), with a loss of amplitude attenuation of the second pulse occurring at intervals greater than 100ms (17, 19).

Other than the paired pulse paradigm, response latencies were also used to indirectly evaluate stimulation of dorsal afferents to trigger a reflex response (23), along with the use of vibration to demonstrate pre-synaptic inhibition of motor responses (14).

Outcome measurement

There were a large variety of outcome measures employed by therapeutic investigations to evaluate motor performance, with 27 different measures used across 14 investigations (Table 8). A total of 8 studies measured joint kinematics, 5 studies assessed functional outcomes and 4 studies assessed gait parameters and force production. Only one therapeutic investigation evaluated the effects of tSCS on subjective quality of life outcomes (35). Apart from the recording of EMG data (n=22), the most frequently employed objective outcomes in therapeutic studies were an evaluation of AIS scoring (n=5), goniometer data of joint angles (n=4), pressure/loading force plate data (n=4) and a measure of gait cycle duration (n=3).

Neurophysiological investigations focused primarily on objectively evaluating the amplitude of EMG responses evoked from tSCS, although some studies additionally looked at the conditioning effects of tSCS on spinal excitability as measured by H-reflex and M-wave amplitude (15, 17, 20, 26). Temporal/phasic modulation of responses evoked by tSCS during gait were also assessed by one study (14).

Table 8. Outcomes selected in the included studies evaluating the effects of therapeutic transcutaneous spinal cord stimulation in spinal cord injured individuals

Study	Force	Kinematics	Gait	Function	Other
Hofstoetter *et al.*, 2013 (6)	-	joint angles (goniometer)	Stride length, cycle duration (pressure switches)	-	-
Gad *et al.*, 2015 (57)	-	-	-	-	Robotic assistance
Gerasimenko *et al.*, 2015 (38)	-	joint angles (goniometer)	-	-	-
Hofstoetter *et al.*, 2015 (18)	-	joint angles (goniometer)	Swing/stance phase duration, cycle duration (foot sensor)	-	-
Bedi, 2016 (58)	-	-	-	-	-
Minassian *et al.*, 2016 (19)	-	-	-	-	-
Gad *et al.*, 2017 (41)	-	joint angles (goniometer and EKSO position sensors)	cycle duration (EKSO device)	-	Self-scoring: muscle tone, sensation, perspiration, coordination, level of robotic assistance, mean HR/BP during training
Freyvert *et al.*, 2018 (44)	Handgrip force measurement	-	-	UEMS (AIS), ARAT	Spasticity (MAS)
Gad *et al.*, 2018 (43)	Handgrip force measurement (transducer)	-	-	Motor and sensory scores (AIS)	Self-report QoL
Inanici *et al.*, 2018 (35)	Pinch strength (pinch gauge)	-	-	AIS scoring, GRASSP	QoL questionnaires (WHO Quality of Life - BREF, SF-Qualiveen, SCIM III)
Rath *et al.*, 2018 (36)	-	Video and 3D kinematic recordings (Xbox One Kinect), centre of pressure (force plate system)	-	-	-
Sayenko *et al.*, 2019 (42)	Knee assistance (force sensing resistor)	Centre of pressure (force plate)	-	-	Qualitative level of assistance, time spent standing
Alam *et al.*, 2020 (40)	-	joint angles and body positions (integrated motion capture system), Sit-to-stand transitions (force plate)	-	AIS scoring	-
Shapkova *et al.*, 2020 (37)	-	Joint angles and body position (ExoAtlet Global exoskeleton), foot loading (force plates and F-Scan sensors)	Hauser Ambulation Index, maximum nonstop walk duration (ExoAtlet Global exoskeleton), Asymmetry Index (ASI)	AIS scoring	Spasticity (MAS), spinal excitability (H-Reflex amplitude)

Table 8: Outcomes measures employed by therapeutic studies investigating the outcomes of tSCS on motor rehabilitation in individuals with SCI. ***Abbreviations:*** *AIS; ASIA Impairment Scale, ARAT; Action Research Arm Test, ASI; asymmetry index, EKSO; Ekso Bionics, EMG; electromyography, GRASSP; Graded and Redefined Assessment of Strength, Sensibility and Prehension, MAS; Modified Ashworth Scale, QoL; quality of life, SCIM III; Spinal Cord Independence Measure Version III, SF; short form, UEMS; upper extremity motor score, WHO; World Health Organisation.*

Surface Electromyography

All studies reported the use of surface EMG to evaluate motor responses. Recordings from over 24 different muscle locations on the lower limb (n=11), upper limb (n=9), and trunk (n=4) were described. An overview of the recording, processing and presentation of EMG signals are presented in Tables 9 and 10 for neurophysiological and therapeutic studies, respectively. Only 7 studies provided adequate details of the preparation including skin preparation, electrode type, shape, composition and inter-electrode distance (6, 14, 16-19, 58). When described, each experiment recorded the use of round silver- silver chloride electrodes with an interelectrode distance of 1.7, 2 or 3 cm. Sampling frequencies ranged from 1,000 to 10,000 Hz.

Several studies explicitly reported filters for stimulus artefact such as bandpass (38), Butterworth (6, 14, 41) or linear adaptive filters (36, 42), whereas others attempted to quantify stimulation artifact by placing electrodes on alternative trunk muscles that were not directly stimulated and using this data to then inverse filter surface EMG signal channels (16, 18, 19). The most popular methods for EMG amplitude processing were the use of full-wave rectification (20, 36, 42), the root mean square (6, 19, 58) and integrated EMG value (35, 41, 43). Several studies chose only to present raw dynamic EMG data (6, 38, 40).

Only a small number of studies normalised EMG amplitude, three of which were therapeutic investigations (18, 42, 43) and six of which were neurophysiological investigations (14, 16, 17, 20, 23, 26). Evoked responses were typically normalised to maximal response at a specific stimulus intensity (20, 26, 42) or when evaluating PAD, the amplitude of the second stimulus of a pair was normalised relative the first (17, 20, 23). A single investigation recording evoked potentials (14), temporally normalised the responses of pulsed stimulus using foot switch pressure sensors in an attempt to evaluate if the spinal cord could modulate the evoked

responses based on the phase of gait. The majority of therapeutic studies recorded dynamic EMG during voluntary movements. However, these signals remained for the most part un-normalised, often with the presentation of exemplary un-rectified EMG traces.

Table 9. EMG recording and signal processing for studies carrying out neurophysiological assessments

PREPARATION/RECORDING			SIGNAL PROCESSING				RESULTS
**Study**	**Muscles [Preparation Described]**	**Recording Device [Sampling Frequency]**	**Filter Passband [Stim artefact filtering]**	**Rectification**	**Cycle averaging**	**Amplitude Normalization**	**Output Presented**
Dy *et al*., 2010 (14)	Sol, MG, TA, med hams, VL [✓]	Hard wired A/D board and customized labVIEW software [200Hz]	20-1000Hz for resting and standing 40-500Hz for stepping [✓]	P2P amplitude for resting standing. Full wave rectified and peak for stepping.	12 MMR responses	Mean MMR for each muscle was normalized to sol responses as stim electrode placement was determined by optimization of sol response	Quantitative comparison between NI and SCI for MMR resting and standing, and phase-dependant MMR during stepping
Emeliannikov *et al*., 2016 (15)	RF, BF, TA, and LG [✗]	Viasys Viking Select [NS]	- [✗]	P2P amplitude	-	-	Comparison of MMR, H-Reflex and M-Wave at rest.
Hofstoetter *et al*., 2018 (16)	RF, BF, TA and TS [✓]	1) DasyLab 11.0 2) Codas ADC system [2048 and 2002Hz]	1) 10-500Hz 2) 30-700Hz with add. 500Hz low-pass [✓]	P2P amplitude	-	-	Quantitative comparison of 1st and 2nd MMR amplitude for TSS and ESS. Onset offset and duration of 1st and 2nd MMR responses. Normalised response thresholds for TSS and ESS
Hofstoetter *et al*., 2019 (17)	RF, BF, TA and Sol [✓]	Phoenix multichannel EMG system [2048Hz]	10-1000Hz [✓]	P2P amplitude	10	Response amplitude of 2nd stimulus in each pair was normalized to the respective 1st for increasing inter-pulse interval (20-5000ms)	Quantitative comparison between NI and SCI for recovery of 2nd PRM as inter-pulse interval increased.
Murray and Knikou, 2019 (20)	Sol, MG, PL, TA, med hams, lat ham, RF, and GRC [✗]	1401 Plus System [2000Hz]	10-1000Hz [✗]	Full wave rectified AUC for each TEP response.	15	A. Responses at increasing intensities were normalized to the associated max response for recruitment curve. B. Responses at increasing frequency (0.1 – 1.0 Hz) normalised to response at 0.1Hz for HD.	Quantitative comparison of recruitment curve sigmoid parameters, PAD and HD, before and after 60min TSS.

						C. Response amplitude of 2nd stimulus of a pair was normalized to the respective 1st for PAD	
Atkinson *et al.*, 2020 (26)	RF, VL, med ham, TA, MG, Sol. [✗]	MA300 EMG System [5000Hz]	- [✗]	P2P amplitude	10	Recruitment responses normalized to P2P amplitude at the maximum rate of recruitment (RRmax) within each muscle. CTI: 2nd stimulus in each pair was normalized to the respective 1st for increasing inter-pulse interval (40-160ms)	Quantitative comparison of interlimb conditioning between NI and SCI.
Militskova *et al.*, 2020 (25)	RF, med ham, TA, sol [✗]	Neuro MEP- (Neurosoft, Ivanovo, Russia) [5000Hz]	- [✗]	-	10	-	Quantitative comparison of SEP latency, threshold and amplitude across (A) 3 stim sites, (B) lying supine vs. standing and (C) pre-post-rehab
Wu *et al.*, 2020 (23)	APB, ADM, FCR, BB [✗]	Customized LabVIEW software (National Instruments USB-6363) [5000Hz]	15-2000Hz [✗]	-	10	Response amplitude of 2nd stimulus of a pair was normalized to the respective 1st (PAD)	Quantitative comparison recruitment curves across stim configuration. Quantitative comparison of PAD across stim intensity between NI and SCI.

Table 9. A summary of evoked surface EMG data collection, recording and signal processing. Abbreviations: ADM; abductor digiti minimi, APB; abductor pollicis brevis, BB; biceps brachii, AUC; area under curve, BR; brachioradialis, CTI; conditioning-test interval, Delt; deltoid, DGO; ED; extensor digitorum, FCR; flexor carpi radialis, FD; flexor digitorum, GRC; gracilis, ham; hamstrings, HD; homosynaptic depression, lat ham; lateral hamstrings, LG; lateral gastrocnemius, med ham; medial hamstrings, MG; medial gastrocnemius, MMR; multisegmental monosynaptic response, P2P; peak-to-peak, PAD; post-activation depression, PL; peroneus longus, Q; quadriceps, RA; rectus abdominis, RF; rectus femoris, Sol; soleus, SEP; spinally evoked potential, TA; Tibialis Anterior, TB; triceps brachii, TFL; tensor fascia lata, TP; tibialis posterior, TS; triceps surae/calf, VL; vastus lateralis

**Preparation described refers to a clear description of preparation of the skin before surface electrode application, recording electrode type, orientation, shape and composition as well as interelectrode distance.*

†Artifact filtering refers to an attempt made by the authors to account for and remove artifacts contaminating or obscuring the recorded EMG signals such as with the use of a filter.

Table 10. EMG recording and signal processing for therapeutic studies

Study	PREPARATION/RECORDING		SIGNAL PROCESSING				RESULTS
	Muscles [Preparation Described]	Recording Device [Sampling Frequency]	Filter Passband [Stim artefact filtration]	Rectification	Cycle averaging	Amplitude Normalization	Output Presented
Hofstoetter *et al.*, 2013 (6)	Q, Ham, TA, TS [✓]	Wired EMS Handels system [2048Hz]	10 - 500 Hz [✗]	Raw EMG	-	-	Exemplary raw EMG traces during stepping. Qualitative comparison of stim on/off
Gad *et al.*, 2015 (57)	Sol, MG, TA, RF, VL. [✗]	Wired A/D board and customized labVIEW software [10,000Hz]	10 – 5000 Hz [✗]	Integrated (presented in Fig 2)	30 steps	-	Exemplary rectified and integrated EMG averaged over 30 steps. Exemplary raw EMG during voluntary movement.
Gerasimenko *et al.*, 2015 (38)	Sol, MG, TA, med ham, VL [✗]	Wired A/D board and customized labVIEW software [10,000Hz]	10-10,000Hz [✓]	-	-	-	Exemplary raw EMG during voluntary movement. Scatter-plot of antagonistic muscle activity patterns. Qualitative description of EMG change during stim.
Hofstoetter *et al.*, 2015 (18)	Q, Ham, TA, TS [✓]	EMS-Handels system [2048Hz]	10 -500 Hz [✓]	RMS	10 gait cycles	EMG during stance and swing phase normalised to muscle activity with stim off	Exemplary raw EMG during stepping. Radar chart of RMS during stance and swing. Qualitative comparison of stim on/off
Bedi, 2016 (58)	Q, Ham, TA, TP [✓]	Neurostim Medicad System [NS]	10 - 500 Hz [✗]	RMS	3 repetitions per side	-	Tables of RMS data during voluntary movement before and after stim
Minassian *et al.*, 2016 (19)	Q, Ham, TA, TS, TFL [✓]	Wired Poly-EMG system (EMS-Handels) [2048Hz]	20 - 500 Hz [✓]	RMS	10 gait cycles	-	Within subject quantitative comparison (ANOVA) of RMS data for treadmill speed x hip extension. Exemplary raw EMG during stepping and standing.
Gad *et al.*, 2017 (41)	BB, FD, ED [✓]	Wired Powerlab and LabChart. Delsys EMG System also mentioned. [10,000Hz]	10 -1000 Hz [✓]	Integrated	30 steps 5 evoked responses	-	Exemplary raw EMG and iEMG during stepping and voluntary movement. Exemplary evoked responses. Qualitative comparison of EMG during passive and active stepping during the intervention.

Freyvert *et al*,. 2018 (44)	FD, ED, BR, BB, TB [✗]	Konigsberg EMG system [NS]	- [✗]	-	9 x 3.5 sec hand-grip Repetition	-	Quantitative comparison of raw EMG amplitude across each test phase Exemplary raw EMG during voluntary hand grip tasks.
Rath *et al*., 2018 (36)	RA, Obl, E-T7, E-L3, RF, delt [✗]	Wired Powerlab 16/35 [2000Hz]	10 -2000 Hz [✓]	Full wave rectification	-	-	Exemplary rectified EMG during trunk movement. Quantitative comparison of mean EMG with stim on/off.
Gad *et al*., 2018 (43)	BB, FD, ED [✗]	Wired Powerlab [10,000Hz]	10-10,000Hz, 60Hz Notch [✗]	Integrated	5 evoked responses per intensity	Evoked responses normalised to baseline.	Exemplary raw and iEMG of evoked responses. Exemplary raw EMG traces during voluntary contractions. Quantitative comparison of iEMG response to stim on/off and pre- post-intervention
Inanici *et al*., 2018 (35)	Delt, TB, BB, BR, ED, FD, ADM, thenar muscles [✗]	Wired Delsys Bagnoli system [1000Hz]	- [✗]	Raw EMG	-	-	Examplary trace of evoked response in thenar muscles compared every 2 weeks across intervention.
Sayenko *et al*., 2019 (42)	Sol, TA, VL, med ham [✗]	Wired Powerlab [4000Hz]	10 -2000 Hz [✓]	Full wave rectification and RMS	6 evoked responses	EMG during anterior/posterior weight shift normalised to activity during initial position	Exemplary raw EMG during transition and standing. Quantitative comparison of mean EMG between stim on/off, sitting and standing and Quantitative comparison of RMS EMG and motor thresholds across training sessions.
Alam *et al*., 2020 (40)	Q, TA, ham, gastric [✗]	Wireless, BTS Telemg [2000Hz]	[✗]	P2P amplitude of MEPs.	-	-	Exemplary raw EMG during sit-to-stand and stand-to-sit task. Intensity response curves for each muscle.
Shapkova *et al*., 2020 (37)	RF, BF, GL, TA [✗]	Viasys Viking Select [2000Hz]	- [✗]	-	-	-	Exemplary trace of H-Reflex and MMR reseponse.

Table 10. A summary of dynamic surface EMG data collection, recording and signal processing. ***Abbreviations:*** *BB; biceps brachii, delt; deltoid, ED; extensor digitorum, E-L3; erector spinae at level of L3, EMG; electromyography, E-T7; erector spinae at level of T7, FD; flexor digitorum, Ham; hamstrings, iEMG; integrated EMG, med ham; medial hamstrings, MG; medial gastrocnemius, obl; external oblique, P2P; peak-to-peak, PL; Peroneus Longus, Q; quadriceps, RA; rectus abdominis, RF; rectus femoris, RMS; root mean square, sol; soleus, TA; Tibialis Anterior, TB; triceps brachii, TFL; tensor fascia lata, TP; tibialis posterior, TS; triceps surae, VL; vastus lateralis.*

** Recording electrodes described refers to a clear description of preparation of the skin before surface electrode application, recording electrode type, orientation, shape and composition as well as interelectrode distance.*

† Artifact filtering refers to an attempt made by the authors to account for and remove artifacts contaminating or obscuring the recorded EMG signals such as with the use of a filter or reference EMG electrodes for artefact cancellation

Safety and adverse events

Of all 22 studies included in this review, only 5 explicitly reported on the presence or absence of adverse events (20, 23, 35, 42, 43). While some studies made comments on stimulation tolerability and pain levels (18, 37, 40, 44, 57), there were insufficient details to rule out all potential safety issues or complications. Three studies reported the complete absence of adverse events while monitoring vital signs throughout, (20, 35, 43). The recorded events from the two other studies included: a modest increase in tone in the 24hrs post treatment, unintentional activation of the micturition reflex and voiding during standing, skin breakage and transient redness (42), as well as discomfort during stimulation at high intensities, asymptomatic variations to heart rate and blood pressure and mild side effects possibly related to cervical stimulation including incidents of light headedness, feeling flushed, nausea, a metallic taste, a sensation of 'sharp' breathing, neck pain, and throat discomfort (23). None of the adverse events recorded were reported to be consistent across treatment sessions, serious or long-lasting.

Discussion

Summary of findings

Since the introduction of tSCS, the number of investigations into the neurophysiological properties and clinical effects have increased, and there is much need for studies that provide greater insights into the functionality of this method. This review separated studies utilising tSCS into two broad categories; studies using the method to evaluate neurophysiological properties of the spinal circuitry and those using the technique as a therapeutic modality. While publications in both categories have grown in number, the quality of the current evidence base is limited, and a large degree of methodological heterogeneity exists between studies. In particular, extensive variability in stimulation parameters and inconsistent processing and/or presentation of electromyographic signals make it difficult to draw meaningful conclusions about the therapeutic effect of tSCS on motor engagement. Efforts should be made in future studies to standardise reporting of muscle activity as well as the electrical parameters of tSCS being administered including electrode dimensions and location, charge polarity, phase duration and stimulation frequency.

A comparison between neurophysiological and therapeutic investigations

Thus far, neurophysiological investigations have focused on the production of evoked motor potentials and the properties of these responses, such as factors affecting response modulation (14, 25, 26) and the characteristics and reflex contributions of stimulation responses (16, 17, 20). In evaluating the electrophysiological impact of tSCS, these experiments can improve our understanding of stimulating spinal circuitries and explore the effects of different parameters and other variables. In neurophysiological investigations, stimulation is generally applied with individual or paired pulses at low frequencies in order to evaluate an evoked response or attain a motor threshold. As such, eliciting specific PRM reflexes is the likely target of these investigations and the stimulation parameters are selected accordingly. Indeed, all neurophysiological investigations recorded the reflex origin of evoked responses. Despite the

shorter experiment duration, this review found less studies investigating the neurophysiological properties of tSCS compared to the therapeutic outcomes.

In contrast, clinical therapeutic investigations aim to exploit tSCS in order to neuromodulate the spinal cord and augment motor responses produced by individuals with SCI (46). As a result, tSCS is generally applied for a longer duration and paired with specific rehabilitative activities. Only two therapeutic investigations (18, 19) attempted to quantify the nature of motor responses generated by tSCS (afferent vs. efferent stimulation). Spinal stimulation has been suggested to produce excitatory input and activate systems such as the PSS and CPGs in order to reduce the firing thresholds required to propagate signals and produce voluntary movement (29, 30). It is likely that these aims are considered in the selection of stimulation parameters and may explain why several therapeutic studies have chosen large stimulation electrodes (58) or multiple stimulation sites (35, 36, 38, 40-43, 57), as opposed to targeting specific sites and spinal levels as is typically carried out in neurophysiological assessment.

Quality of included trials

Research investigating the effects of tSCS is an emerging field that predominantly consists of exploratory clinical trials and studies were unsurprisingly found to be of poor-to-fair quality using the D&B Checklist. Sample sizes were generally small, and 7 of the therapeutic studies were single participant case reports. In recent years sample sizes have grown, with several studies published in 2019 and 2020 scoring on the B&D Checklist between 1 – 5 points from retrospective power analyses (17, 20, 23, 37, 42).

All studies scored poorly on external validity due to a lack of balanced protocols and reporting on recruitment methods. Research in this field is difficult to extrapolate to the population as a whole, as people with SCI differ markedly, even within the same clinical classifications (59). Previous studies in this population have attempted to employ balanced protocols with respect to variables such as AIS classification (59). However, similar designs have not yet been used

to explore the effects of tSCS. Moreover, no included study provided a comprehensive account of their recruitment protocols and only 6 detailed eligibility criteria, (20, 23, 37, 42-44). Regardless of inherent recruitment challenges, greater transparency is needed.

Finally, studies scored poorly for internal validity and there was limited use of randomisation, blinding or sham stimulation. The use of non-randomized designs is common in studying individuals with chronic conditions such as SCI due to inherent methodological, ethical, and practical considerations (60, 61). Despite this, two studies did employ randomisation in their crossover design (36, 42). In only three studies were assessors blinded to the intervention (37, 42, 44) and Sayenko *et al.* (42) was the only study that attempted to use a placebo in the form of two sham stimulation conditions; one on a different location on the spinal cord that did not project to the motor pools assessed and another designed to give the sensation of stimulation without targeting motor responses. While these forms of sham stimulation may not be completely inert in their effects, it demonstrates the only attempt to account for the potential placebo effect of stimulation.

Stimulation parameters

Electrode polarity and configuration

The stimulation parameters and, in particular, the electrode configurations play an important role in determining the electrical field that is produced by tSCS and, as a result, the structures that are targeted within the field. One of the criticisms of tSCS, when compared to the epidural alternative, is failure to create a localised electrical field thereby limiting activation selectivity (16, 62). This review found a lack of consensus regarding electrode configurations, particularly in the cervical region, and limited rationale for selected configurations.

All included studies either placed the cathode posteriorly over the spinal column or used biphasic current with alternating polarity. Other studies investigating use of tSCS on neurologically intact individuals placed the anode posteriorly (6, 16-18, 23, 35, 43), but this has

not yet been tested in subjects with SCI. The majority of studies use monophasic current with only a limited number employing biphasic current (6, 16-18, 23, 35, 43). Biphasic current has been noted to reduce risk of tissue damage (63), and Hofstoetter *et al.* (16) found that evoked responses were initiated by the abrupt change of polarity of the biphasic stimulation pulses. Only one included study (23) directly tested different pulse conditions and found biphasic 2-ms or monophasic 1-ms pulses, with the cathode posterior, elicited larger responses at lower intensities.

The application of stimulating electrodes varies throughout included studies, with regard to both rostro-caudal and mediolateral alignment. The cathodes were positioned paravertebrally or centrally over the midline but, thus far, no conclusions have been drawn on the effects of these different configuration. One study in uninjured individuals demonstrated that lateralisation of motor responses in lower limbs (i.e. right/left differentiation) can be achieved from the placement of stimulating electrodes ~2cm laterally from the lumbar spinous process (64). With regard to the spinal levels selected, differential activation of muscle responses has been demonstrated from stimulation of different points along the rostro-caudal axis of the lumbosacral enlargement in neurologically intact (65, 66) and injured (25) individuals.

Several therapeutic studies in this review found superior effects from stimulating the coccygeal level along with a lumbar stimulation site, however the rationale of combining neuromodulation of structurally different areas remains unclear. By stimulating the cauda equina, PRM reflexes can be distinguished from direct motor responses by their different latencies, reflecting the different lengths of the neural pathways, with decreasing latencies for increased stimulation intensities (2, 67). Indeed, for the cathode position, Roy *et al.* (69) found, using a paired pulse stimulation test, that spinal reflexes were optimally elicited with tSCS when the cathode was over the upper-lumber vertebrae (L1-L3), and M-waves were optimally elicited with tSCS when the cathode was placed more caudally (L5, S1). If the proposed mechanism for tSCS involves activation of spinal reflex pathways to lower threshold for CPG or voluntary movement, then it

would seem important that therapists confirm that the stimulus is transpinally modulated and not just acting as surrogate FES.

One study tested for optimal evoked responses at different spinal levels prior to commencing the experimental protocol in order to account for inter-individual variability (26). The consideration of participant-specific parameter selection could better account for anatomical variation between individuals (26, 70), such as conus medullaris termination level (71), or factors such as injury scar tissue thereby providing more targeted treatment.

For the stimulation of lower limb responses, the anode was placed over the lower abdomen (6, 16-19, 25, 37), anterior superior iliac spine (26), or iliac crests (14, 20, 38, 40, 41). This is consistent with other previous investigations testing neurologically intact participants (21, 66, 72, 73). In the cervical region, there was greater variance as anodes were placed superiorly above the sternal notch (23) or inferiorly on the iliac crest (35, 41) and ASIS (44). Similarly, in cervical tSCS studies of uninjured individuals, anode locations vary between the left acromion (74), upper trapezius and mid clavicle (75), and the midline over the anterior neck (22). A previous study investigating the effects of anode position have shown that it is critical for inducing spinal reflexes (76). Limited human research has explored the effects of different anode-cathode configurations as a determinant for stimulation outcome. This is spite of modelling studies of lumbar tSCS which have positioned the reference electrode centrally over the abdomen (7, 8) and noted that excitation "hot spots" depended on the position of the posterior root fiber with respect to the stimulating electrodes (8).

Electrical characteristics

The voltage that builds up on a skin electrode during a pulse depends on the charge density, i.e., the accumulated charge divided by the electrode area. Large electrodes have lower charge density and therefore lower pulse voltages for the same current. Vargas Luna *et al.* (77) define a charge density threshold beyond which electro-osmotic effects become significant in the skin

conduction mechanism. This may have implications for skin comfort and irritation. The charge density in the reviewed studies ranged between 1.8 to 39 μC/cm^2. Large electrodes also disperse the current in the underlying tissues which may reduce the likelihood of reaching stimulation thresholds at target neurons while increasing the probability of unwanted collateral stimulation.

For sustained trains of pulses lasting several seconds the accumulated direct current, stimulation of pain receptors and heating effect in the skin must be considered. Monophasic pulse trains produce direct current which can give rise to unwanted electrochemical effects at the electrode site leading to skin irritation and even damage. DC levels higher than 0.5 mA/cm^2 at the cathode, and 1.0 mA/cm^2 at the anode, are potentially harmful (78). Even for balanced biphasic waveforms, safety standards such as IEC 60601-2-10 require that the user be advised when the skin current density exceeds 2 mA/cm^2. Neurologically intact subjects with normal skin sensation will usually find this current density quite uncomfortable, and even further rigour must be taken by researchers when working with participants with impaired sensation. FDA guidance documents advise against power densities greater than 0.25W/cm^2 due to the potential heat damage to tissue.

Position of participants

The conditions under which stimulation is implemented have been reported to influence motor response outcomes and studies have demonstrated that spinally-evoked muscle response amplitudes are facilitated or supressed depending on positional factors and activity phases (5, 14, 79). The majority of included therapeutic studies targeting lower limb responses were partly conducted in an upright position (6, 18, 19, 37, 40-42, 57), whereas neurophysiological investigations of lower limb responses were predominantly in supine (16, 17, 20, 26) or seated (15, 23). Different testing conditions have the ability to alter the motor responses generated by tSCS and may cause disparity between results from different studies.

A case report by Militskova *et al.* (25) found that spinally evoked response amplitudes were highest in standing, compared to supine, in an individual with SCI. Conversely, a study of 10 healthy participants by Dannet *et al.* (80) found that response amplitudes were higher in supine than standing, but that response thresholds were lowest in standing. These studies suggest that results could be due to position-dependent changes in the electrical field distribution or afferent input altering spinal excitability. Additionally, body position alters the location of the spinal cord within the vertebral canal (81). Thus far, no studies have investigated the effects of positioning on cervicothoracic stimulation and upper limb responses to the authors' knowledge. Future studies must consider the effects of activity and body positioning and explore conditions that mimic potential clinical scenarios.

Considerations in the interpretation of EMG data

The most common objective outcome measure which was used by all studies in this review was motor response recorded via surface EMG. These signals were used either to quantify the evoked responses at rest, or the level of muscle activity recorded during voluntary movement. The reported methods were in most cases lacking in detail and in some cases not reflective of best practice for the recording and processing of surface EMG (82, 83).

In terms of evaluating the magnitude of evoked responses, the majority of studies reported peak-to-peak amplitude of the unrectified EMG signal during a specific time-window after a tSCS pulse was administered. Exceptions to this rule included Murray and Knikou (20) who quantified the area under the curve of the rectified waveforms and Dy *et al.* (14) who were evaluating phase-dependant modulation of the evoked response during stepping. The reflex nature of evoked responses was most commonly evaluated by quantifying PAD. Other neurophysiological indices calculated from these evoked EMG waveforms included latency and motor threshold (typically defined as the minimum current required to elicit a measurable evoked response).

A notable methodological consideration for EMG recorded during tSCS (and eSCS) is the presence of considerable stimulus artefact within the signal. This review identified several filtering approaches including employing high order bandpass filtering of 30-200Hz (38), lower order 6th order Butterworth filters with a passband of 30-1000Hz (41) and the implementation of blanking intervals based on stimulus artefact recorded from trunk musculature (18, 19). Of note was the detailed description by Rath *et al.* (36) of a multi-stage "linear adaptive filter" process which was subsequently utilised by Sayenko *et al*. (42). The efficacy of one approach relative to another for optimising signal:noise ratio has yet to be fully elucidated. Unfortunately, the majority of therapeutic studies (n=8) did not explicitly report any attempt at filtering non-physiological noise associated with tSCS.

A recent consensus statement on EMG signal normalization highlighted its importance for comparing muscle activity between measurement sessions and/or experimental conditions (83). Despite its well-documented importance, we found only two therapeutic studies (18, 42) which described any attempt at normalizing dynamic EMG signals between sessions or experimental conditions. Many of the therapeutic studies present un-normalised and/or un-rectified exemplary EMG traces, providing some limited qualitative evidence of motor engagement during gravity neutral leg movements (38), assisted robotic stepping (57), sit-to-stand movement (40) or voluntary handgrip task (44). Other studies attempted to statistically compare un-normalised EMG signals recorded intermittently over several months (41, 44). In either case, no meaningful conclusions regarding the efficacy of tSCS to alter muscle activity in patients with SCI may be drawn from this data. Future studies attempting to examine the effect of tSCS therapy on muscle activity during dynamic movements are recommended to present normalized EMG envelopes averaged across multiple cycles or repetitions. Examples of this approach can be seen in the detailed qualitative (84) and quantitative (85) comparisons of EMG recorded from patients with SCI during stepping in the presence or absence of eSCS.

Measurement considerations in the evaluation of the motor effects of tSCS

A large number of other outcome measures were used by therapeutic studies investigating the effects of tSCS, whereas neurophysiological investigations focused primarily on electrophysiological measurement of evoked muscle response. Additionally, several studies included reports of non-standardised measures such as level of robotic assistance or qualitative self-report data (41, 42, 57). Despite this variability in selected outcome measures, the majority of studies focused on specific movement-related outcomes, with only a few studies measuring the impact of therapeutic tSCS on an individual's overall functional ability (35, 37, 40, 43, 44) or quality of life (35, 43). The improvement of functional abilities, reflected in activities of daily living, have been noted as the most meaningful and valued outcomes (86). In future, researchers are advised to look toward published consensus guidelines to select outcome variables that are validated for use with SCI populations, such as those recommended by SCOPE, the spinal cord outcomes partnership endeavour (87). By selecting standardised outcome measures that are clinically meaningful, the meta-analysis of results will be possible, and comparisons can be drawn regarding effectiveness to other interventions promoting motor recovery in SCI populations.

Limitations of this review

We acknowledge that this review is subject to several potential limitations. Due to the variance in terminology in this field and the lack of standardised nomenclature, it is possible that relevant studies may have been missed by our search strategy. Additionally, our eligibility criteria specified that only studies including EMG outcomes should be included and therefore other studies detailing the tSCS parameters may have been excluded. Finally, study outcomes were not possible to pool due to the heterogeneity of included experiments, and therefore conclusions regarding the optimal stimulation parameters and study protocols cannot be drawn.

Recommendations and future directions

To fully exploit the capacity of tSCS to generate motor activity, future research must directly explore the effects of different parameters to determine the optimal conditions for desired motor outcomes. Greater justification for the selection of therapeutic stimulation parameters needs to be provided by experiments that bridge the gap in our understanding of parameter optimisation, clinical application and the mechanisms that promote motor recovery. Moreover, tSCS must be evaluated further in under-represented fields such as the upper limbs, trunk and in sub-acute stages of injury.

The quality of future trials would be improved with better reporting of recruitment methods and intervention protocols and with the application of techniques such as randomisation and sham-stimulation. Finally, the presence or absence of adverse events and study limitations must be explicitly detailed to provide a larger evidence base supporting the safety and feasibility. Studies must also increase use of standardised outcome measures that are validated for application in populations with SCI and improve the methodological rigour for data collection, processing and reporting of EMG data to allow for future meta-analysis of results.

Conclusions

The results of this systematic review indicate that studies investigating the effects of tSCS interventions for individuals with SCI face both methodological and measurement deficiencies. While initial investigations have improved our understanding of the neurophysiological impact of this technology and demonstrated its feasibility in motor rehabilitation, greater homogeneity in the reporting of stimulation parameters and outcome measurement will be required to pool cumulative outcomes from small sample sizes. A higher quality of studies will be needed to demonstrate conclusive evidence on the standardised application and uses of tSCS.

Acknowledgments (Funding)
This study was supported by the Disruptive Technology Innovation Fund, grant number DT - 2018-0128

Competing Interests: The author has declared that no competing interests exist.

Supporting Information

S1 File. Keywords and search criteria

Author Contributions

Conceptualization: Clare Taylor, Conor McHugh, Conor Minogue, Neil Fleming.

Data curation: Clare Taylor, Conor McHugh, Neil Fleming.

Formal analysis: Clare Taylor, Conor McHugh, Conor Minogue, Neil Fleming, David Mockler, Richard Reilly.

Funding acquisition: Neil Fleming, Richard Reilly, Conor Minogue.

Investigation: Clare Taylor, Conor McHugh, Neil Fleming.

Methodology: Clare Taylor, Conor McHugh, Neil Fleming, Conor Minogue, David Mockler.

Project administration: Clare Taylor

Supervision: Neil Fleming, Richard Reilly.

Validation: Conor McHugh, Neil Fleming.

Writing – original draft: Clare Taylor

Writing – review & editing: Clare Taylor, Conor McHugh, Neil Fleming, Conor Minogue, Richard Reilly.

References

1. Gerasimenko Y, Gorodnichev R, Moshonkina T, Sayenko D, Gad P, Reggie Edgerton V. Transcutaneous electrical spinal-cord stimulation in humans. Annals of Physical & Rehabilitation Medicine. 2015;58(4):225-31.
2. Minassian K, Persy I, Rattay F, Dimitrijevic MR, Hofer C, Kern H. Posterior root-muscle reflexes elicited by transcutaneous stimulation of the human lumbosacral cord. Muscle and Nerve. 2007;35(3):327-36.
3. Minassian K, Persy I, Rattay F, Pinter MM, Kern H, Dimitrijevic MR. Human lumbar cord circuitries can be activated by extrinsic tonic input to generate locomotor-like activity. Human Movement Science. 2007;26(2):275-95.
4. Hofstoetter US, Minassian K, Hofer C, Mayr W, Rattay F, Dimitrijevic MR. Modification of reflex responses to lumbar posterior root stimulation by motor tasks in healthy subjects. Artif Organs. 2008;32(8):644-8.
5. Courtine G, Harkema SJ, Dy CJ, Gerasimenko YP, Dyhre-Poulsen P. Modulation of multisegmental monosynaptic responses in a variety of leg muscles during walking and running in humans. J Physiol. 2007;582(Pt 3):1125-39.
6. Hofstoetter US, Hofer C, Kern H, Danner SM, Mayr W, Dimitrijevic MR, et al. Effects of transcutaneous spinal cord stimulation on voluntary locomotor activity in an incomplete spinal cord injured individual. Biomed Tech (Berl). 2013;58 Suppl 1.
7. Danner SM, Hofstoetter US, Ladenbauer J, Rattay F, Minassian K. Can the human lumbar posterior columns be stimulated by transcutaneous spinal cord stimulation? A modeling study. Artif Organs. 2011;35(3):257-62.
8. Ladenbauer J, Minassian K, Hofstoetter U, Dimitrijevic M, Rattay F. Stimulation of the Human Lumbar Spinal Cord With Implanted and Surface Electrodes: A Computer Simulation Study. Neural Systems and Rehabilitation Engineering, IEEE Transactions on. 2010;18:637-45.
9. Minassian K, Persy I, Rattay F, Dimitrijevic MR, Hofer C, Kern H. Posterior root-muscle reflexes elicited by transcutaneous stimulation of the human lumbosacral cord. Muscle & nerve. 2007;35(3):327-36.
10. Knikou M. Neurophysiological characterization of transpinal evoked potentials in human leg muscles. Bioelectromagnetics. 2013;34(8):630-40.
11. Einhorn J, Li A, Hazan R, Knikou M. Cervicothoracic multisegmental transpinal evoked potentials in humans. PLoS ONE. 2013;8(10):e76940. eCollection 2013 doi: 10.1371/journal.pone.0076940.
12. Krenn M, Hofstoetter US, Danner SM, Minassian K, Mayr W. Multi-Electrode Array for Transcutaneous Lumbar Posterior Root Stimulation. Artif Organs. 2015;39(10):834-40.
13. Sayenko DG, Angeli C, Harkema SJ, Edgerton VR, Gerasimenko YP. Neuromodulation of evoked muscle potentials induced by epidural spinal-cord stimulation in paralyzed individuals. J Neurophysiol. 2014;111(5):1088-99.
14. Dy CJ, Gerasimenko YP, Edgerton VR, Dyhre-Poulsen P, Courtine G, Harkema SJ. Phase-dependent modulation of percutaneously elicited multisegmental muscle responses after spinal cord injury. J Neurophysiol. 2010;103(5):2808-20.
15. Emeliannikov DV, Shapkova EY, Moshonkina TR, Gerasimenko YP. Evaluation of motor neuron excitability in lumbosacral spinal cord: Transcutaneous spinal cord stimulation as compared to H-reflex. Fiziologiia Cheloveka. 2016;42(3):32-6.
16. Hofstoetter US, Freundl B, Binder H, Minassian K. Common neural structures activated by epidural and transcutaneous lumbar spinal cord stimulation: Elicitation of posterior root-muscle reflexes. PLoS One. 2018;13(1):e0192013. doi: 10.1371/journal.pone.0192013
17. Hofstoetter US, Freundl B, Binder H, Minassian K. Recovery cycles of posterior root-muscle reflexes evoked by transcutaneous spinal cord stimulation and of the H reflex in individuals with intact and injured spinal cord. PLoS ONE. 2019;14(12):e0227057. doi: 10.1371/journal.pone.0227057
18. Hofstoetter US, Krenn M, Danner SM, Hofer C, Kern H, McKay WB, et al. Augmentation of Voluntary Locomotor Activity by Transcutaneous Spinal Cord Stimulation in Motor-Incomplete Spinal Cord-Injured Individuals. Artif Organs. 2015;39(10):E176-86. doi: 10.1111/aor.12615

19. Minassian K, Hofstoetter US, Danner SM, Mayr W, Bruce JA, McKay WB, et al. Spinal Rhythm Generation by Step-Induced Feedback and Transcutaneous Posterior Root Stimulation in Complete Spinal Cord-Injured Individuals. Neurorehabil Neural Repair. 2016;30(3):233-43.
20. Murray LM, Knikou M. Transspinal stimulation increases motoneuron output of multiple segments in human spinal cord injury. PLoS ONE. 2019;14(3):e0213696. doi: 10.1371/journal.pone.0213696
21. Andrews JC, Stein RB, Roy FD. Post-activation depression in the human soleus muscle using peripheral nerve and transcutaneous spinal stimulation. Neuroscience Letters. 2015;589:144-9.
22. Milosevic M, Masugi Y, Sasaki A, Sayenko DG, Nakazawa K. On the reflex mechanisms of cervical transcutaneous spinal cord stimulation in human subjects. J Neurophysiol. 2019;121(5):1672-9.
23. Wu YK, Levine JM, Wecht JR, Maher MT, LiMonta JM, Saeed S, et al. Posteroanterior cervical transcutaneous spinal stimulation targets ventral and dorsal nerve roots. Clin Neurophysiol. 2020;131(2):451-60.
24. Zheng Y, Hu X. Muscle activation pattern elicited through transcutaneous stimulation near the cervical spinal cord. J Neural Eng. 2020;17(1):016064.
25. Militskova A, Mukhametova E, Fatykhova E, Sharifullin S, Cuellar CA, Calvert JS, et al. Supraspinal and Afferent Signaling Facilitate Spinal Sensorimotor Network Excitability After Discomplete Spinal Cord Injury: A Case Report. Front Neurosci. 2020;14:552.
26. Atkinson DA, Sayenko DG, D'Amico JM, Mink A, Lorenz DJ, Gerasimenko YP, et al. Interlimb conditioning of lumbosacral spinally evoked motor responses after spinal cord injury. Clin Neurophysiol. 2020;131(7):1519-32.
27. Angeli CA, Edgerton VR, Gerasimenko YP, Harkema SJ. Altering spinal cord excitability enables voluntary movements after chronic complete paralysis in humans. Brain. 2014;137(Pt 5):1394-409.
28. Young W. Electrical stimulation and motor recovery. Cell Transplant. 2015;24(3):429-46.
29. Taccola G, Sayenko D, Gad P, Gerasimenko Y, Edgerton VR. And yet it moves: Recovery of volitional control after spinal cord injury. Prog Neurobiol. 2018;160:64-81.
30. Hofstoetter US, Knikou M, Guertin PA, Minassian K. Probing the Human Spinal Locomotor Circuits by Phasic Step-Induced Feedback and by Tonic Electrical and Pharmacological Neuromodulation. Curr Pharm Des. 2017;23(12):1805-20.
31. Minassian K, Hofstoetter US, Dzeladini F, Guertin PA, Ijspeert A. The Human Central Pattern Generator for Locomotion: Does It Exist and Contribute to Walking? Neuroscientist. 2017;23(6):649-63.
32. Dietz V, Fouad K, Bastiaanse CM. Neuronal coordination of arm and leg movements during human locomotion. Eur J Neurosci. 2001;14(11):1906-14.
33. Laliberte AM, Goltash S, Lalonde NR, Bui TV. Propriospinal Neurons: Essential Elements of Locomotor Control in the Intact and Possibly the Injured Spinal Cord. Frontiers in Cellular Neuroscience. 2019;13(512).
34. Eblen-Zajjur AA, Sandkühler J. Synchronicity of nociceptive and non-nociceptive adjacent neurons in the spinal dorsal horn of the rat: stimulus-induced plasticity. Neuroscience. 1997;76(1):39-54.
35. Inanici F, Samejima S, Gad P, Edgerton VR, Hofstetter CP, Moritz CT. Transcutaneous Electrical Spinal Stimulation Promotes Long-Term Recovery of Upper Extremity Function in Chronic Tetraplegia. IEEE Trans Neural Syst Rehabil Eng. 2018;26(6):1272-8.
36. Rath M, Vette AH, Ramasubramaniam S, Li K, Burdick J, Edgerton VR, et al. Trunk Stability Enabled by Noninvasive Spinal Electrical Stimulation after Spinal Cord Injury. J Neurotrauma. 2018;35(21):2540-53.
37. Shapkova EY, Pismennaya EV, Emelyannikov DV, Ivanenko Y. Exoskeleton Walk Training in Paralyzed Individuals Benefits From Transcutaneous Lumbar Cord Tonic Electrical Stimulation. Front Neurosci. 2020;14:416.
38. Gerasimenko YP, Lu DC, Modaber M, Zdunowski S, Gad P, Sayenko DG, et al. Noninvasive Reactivation of Motor Descending Control after Paralysis. J Neurotrauma. 2015;32(24):1968-80.
39. van den Brand R, Mignardot JB, von Zitzewitz J, Le Goff C, Fumeaux N, Wagner F, et al. Neuroprosthetic technologies to augment the impact of neurorehabilitation after spinal cord injury. Ann Phys Rehabil Med. 2015;58(4):232-7.

40. Alam M, Ling YT, Wong AYL, Zhong H, Edgerton VR, Zheng Y-P. Reversing 21 years of chronic paralysis via non-invasive spinal cord neuromodulation: a case study. Annals of clinical and translational neurology. 2020;7(5):829-38.
41. Gad P, Gerasimenko Y, Zdunowski S, Turner A, Sayenko D, Lu DC, et al. Weight Bearing Over-ground Stepping in an Exoskeleton with Non-invasive Spinal Cord Neuromodulation after Motor Complete Paraplegia. Front Neurosci. 2017;11:333.
42. Sayenko DG, Rath M, Ferguson AR, Burdick JW, Havton LA, Edgerton VR, et al. Self-Assisted Standing Enabled by Non-Invasive Spinal Stimulation after Spinal Cord Injury. J Neurotrauma. 2019;36(9):1435-50.
43. Gad P, Lee S, Terrafranca N, Zhong H, Turner A, Gerasimenko Y, et al. Non-Invasive Activation of Cervical Spinal Networks after Severe Paralysis. J Neurotrauma. 2018;35(18):2145-58.
44. Freyvert Y, Yong NA, Morikawa E, Zdunowski S, Sarino ME, Gerasimenko Y, et al. Engaging cervical spinal circuitry with non-invasive spinal stimulation and buspirone to restore hand function in chronic motor complete patients. Sci Rep. 2018;8(1):15546.
45. Tator CH, Minassian K, Mushahwar VK. Spinal cord stimulation: therapeutic benefits and movement generation after spinal cord injury. Handb Clin Neurol. 2012;109:283-96.
46. Megía García A, Serrano-Muñoz D, Taylor J, Avendaño-Coy J, Gómez-Soriano J. Transcutaneous Spinal Cord Stimulation and Motor Rehabilitation in Spinal Cord Injury: A Systematic Review. Neurorehabil Neural Repair. 2020;34(1):3-12.
47. Moher D, Shamseer L, Clarke M, Ghersi D, Liberati A, Petticrew M, et al. Preferred reporting items for systematic review and meta-analysis protocols (PRISMA-P) 2015 statement. Syst Rev. 2015;4(1):1.
48. Downs SH, Black N. The feasibility of creating a checklist for the assessment of the methodological quality both of randomised and non-randomised studies of health care interventions. J Epidemiol Community Health. 1998;52(6):377-84.
49. Lam T, Eng JJ, Wolfe DL, Hsieh JT, Whittaker M. A systematic review of the efficacy of gait rehabilitation strategies for spinal cord injury. Top Spinal Cord Inj Rehabil. 2007;13(1):32-57.
50. Ibitoye MO, Hamzaid NA, Hasnan N, Abdul Wahab AK, Davis GM. Strategies for Rapid Muscle Fatigue Reduction during FES Exercise in Individuals with Spinal Cord Injury: A Systematic Review. PloS one. 2016;11(2):e0149024. doi: 10.1371/journal.pone.0149024
51. Silverman SR, Schertz LA, Yuen HK, Lowman JD, Bickel CS. Systematic review of the methodological quality and outcome measures utilized in exercise interventions for adults with spinal cord injury. Spinal Cord. 2012;50(10):718-27.
52. Eng JJ, Teasell R, Miller WC, Wolfe DL, Townson AF, Aubut JA, et al. Spinal Cord Injury Rehabilitation Evidence: Methods of the SCIRE Systematic Review. Top Spinal Cord Inj Rehabil. 2007;13(1):1-10.
53. Saunders LD, Soomro GM, Buckingham J, Jamtvedt G, Raina P. Assessing the methodological quality of nonrandomized intervention studies. West J Nurs Res. 2003;25(2):223-37.
54. Deeks JJ, Dinnes J, D'Amico R, Sowden AJ, Sakarovitch C, Song F, et al. Evaluating non-randomised intervention studies. Health Technol Assess. 2003;7(27):iii-x, 1-173.
55. Faul F, Erdfelder E, Lang A-G, Buchner A. G*Power 3: A flexible statistical power analysis program for the social, behavioral, and biomedical sciences. Behavior Research Methods. 2007;39(2):175-91.
56. Hamdan PNF, Hamzaid NA, Razak NA, Hasnan N. Contributions of the Cybathlon championship to the literature on functional electrical stimulation cycling among individuals with spinal cord injury: A bibliometric review. Journal of Sport and Health Science. 2020.
57. Gad PN, Gerasimenko YP, Zdunowski S, Sayenko D, Haakana P, Turner A, et al. Iron 'ElectriRx' man: Overground stepping in an exoskeleton combined with noninvasive spinal cord stimulation after paralysis. Conference Proceedings: Annual International Conference of the IEEE Engineering in Medicine & Biology Society. 2015;2015:1124-7.
58. Bedi P. Tapping the Neural Circuitry: Surface Spinal Stimulation in Spinal Cord Injury: a Case Report. Journal of Exercise Science and Physiotherapy. 2016;12.
59. Sherwood AM, McKay WB, Dimitrijević MR. Motor control after spinal cord injury: assessment using surface EMG. Muscle Nerve. 1996;19(8):966-79.
60. Ginis KA, Hicks AL. Exercise research issues in the spinal cord injured population. Exerc Sport Sci Rev. 2005;33(1):49-53.

61. Wilbanks S, Schertz LA, Yuen H, Lowman J, Bickel C. Systematic review of the methodological quality and outcome measures utilized in exercise interventions for adults with spinal cord injury. Spinal cord. 2012;50:718-27.
62. Duffell LD, Donaldson NdN. A Comparison of FES and SCS for Neuroplastic Recovery After SCI: Historical Perspectives and Future Directions. Frontiers in neurology. 2020;11:607-.
63. Hofmann L, Ebert M, Tass P, Hauptmann C. Modified Pulse Shapes for Effective Neural Stimulation. Frontiers in Neuroengineering. 2011;4(9).
64. Calvert JS, Manson GA, Grahn PJ, Sayenko DG. Preferential activation of spinal sensorimotor networks via lateralized transcutaneous spinal stimulation in neurologically intact humans. J Neurophysiol. 2019;122(5):2111-8.
65. Krenn M, Toth A, Danner SM, Hofstoetter US, Minassian K, Mayr W. Selectivity of transcutaneous stimulation of lumbar posterior roots at different spinal levels in humans. Biomed Tech (Berl). 2013;58 Suppl 1.
66. Sayenko DG, Atkinson DA, Dy CJ, Gurley KM, Smith VL, Angeli C, et al. Spinal segment-specific transcutaneous stimulation differentially shapes activation pattern among motor pools in humans. Journal of Applied Physiology. 2015;118(11):1364-74.
67. Zhu Y, Starr A, Haldeman S, Chu JK, Sugerman RA. Soleus H-reflex to S1 nerve root stimulation. Electroencephalogr Clin Neurophysiol. 1998;109(1):10-4.
68. Roy FD, Gibson G, Stein RB. Effect of percutaneous stimulation at different spinal levels on the activation of sensory and motor roots. Exp Brain Res. 2012;223(2):281-9.
69. Roy RR, Harkema SJ, Edgerton VR. Basic concepts of activity-based interventions for improved recovery of motor function after spinal cord injury. Arch Phys Med Rehabil. 2012;93(9):1487-97.
70. Troni W, Di Sapio A, Berra E, Duca S, Merola A, Sperli F, et al. A methodological reappraisal of non invasive high voltage electrical stimulation of lumbosacral nerve roots. Clinical Neurophysiology. 2011;122(10):2071-80.
71. Saifuddin A, Burnett SJ, White J. The variation of position of the conus medullaris in an adult population. A magnetic resonance imaging study. Spine (Phila Pa 1976). 1998;23(13):1452-6.
72. Kaneko N, Masugi Y, Usuda N, Yokoyama H, Nakazawa K. Muscle-Specific Modulation of Spinal Reflexes in Lower-Limb Muscles during Action Observation with and without Motor Imagery of Walking. Brain Sciences. 2019;9(12):21.
73. Kitano K, Koceja DM. Spinal reflex in human lower leg muscles evoked by transcutaneous spinal cord stimulation. J Neurosci Methods. 2009;180(1):111-5.
74. Sabbahi MA, Sengul YS. Cervical multisegmental motor responses in healthy subjects. Spinal Cord. 2012;50(6):432-9.
75. Sabbahi MA, Uzun S, Bittar FO, Sengul Y. Similarities and differences in cervical and thoracolumbar multisegmental motor responses and the combined use for testing spinal circuitries. Journal of Spinal Cord Medicine. 2014;37(4):401-13.
76. Masugi Y, Obata H, Nakazawa K. Effects of anode position on the responses elicited by transcutaneous spinal cord stimulation. Conf Proc IEEE Eng Med Biol Soc. 2017;2017:1114-7.
77. Vargas Luna JL, Krenn M, Cortés Ramírez JA, Mayr W. Dynamic Impedance Model of the Skin-Electrode Interface for Transcutaneous Electrical Stimulation. PLOS ONE. 2015;10(5):e0125609. doi: 10.1371/journal.pone.0125609
78. Bélanger A. Therapeutic Electrophysical Agents: Evidence Behind Practice: Wolters Kluwer Health/Lippincott Williams & Wilkins; 2010.
79. Masugi Y, Kawashima N, Inoue D, Nakazawa K. Effects of movement-related afferent inputs on spinal reflexes evoked by transcutaneous spinal cord stimulation during robot-assisted passive stepping. Neuroscience Letters. 2016;627:100-6.
80. Danner SM, Krenn M, Hofstoetter US, Toth A, Mayr W, Minassian K. Body position influences which neural structures are recruited by lumbar transcutaneous spinal cord stimulation. PLoS ONE. 2016;11(1).
81. Ranger MR, Irwin GJ, Bunbury KM, Peutrell JM. Changing body position alters the location of the spinal cord within the vertebral canal: a magnetic resonance imaging study. Br J Anaesth. 2008;101(6):804-9.
82. Hermens HJ, Freriks B, Disselhorst-Klug C, Rau G. Development of recommendations for SEMG sensors and sensor placement procedures. J Electromyogr Kinesiol. 2000;10(5):361-74.

83. Besomi M, Hodges PW, Clancy EA, Van Dieën J, Hug F, Lowery M, et al. Consensus for experimental design in electromyography (CEDE) project: Amplitude normalization matrix. J Electromyogr Kinesiol. 2020;53:102438.
84. Huang H, He J, Herman R, Carhart MR. Modulation effects of epidural spinal cord stimulation on muscle activities during walking. IEEE Trans Neural Syst Rehabil Eng. 2006;14(1):14-23.
85. Gill ML, Grahn PJ, Calvert JS, Linde MB, Lavrov IA, Strommen JA, et al. Neuromodulation of lumbosacral spinal networks enables independent stepping after complete paraplegia. Nature Medicine. 2018;24(11):1677-82.
86. Steeves JD, Lammertse D, Curt A, Fawcett JW, Tuszynski MH, Ditunno JF, et al. Guidelines for the conduct of clinical trials for spinal cord injury (SCI) as developed by the ICCP panel: clinical trial outcome measures. Spinal Cord. 2007;45(3):206-21.
87. Alexander MS, Anderson KD, Biering-Sorensen F, Blight AR, Brannon R, Bryce TN, et al. Outcome measures in spinal cord injury: recent assessments and recommendations for future directions. Spinal cord. 2009;47(8):582-91.

 Click here to access/download;Figure;Figure 1 Current Waveforms .docx

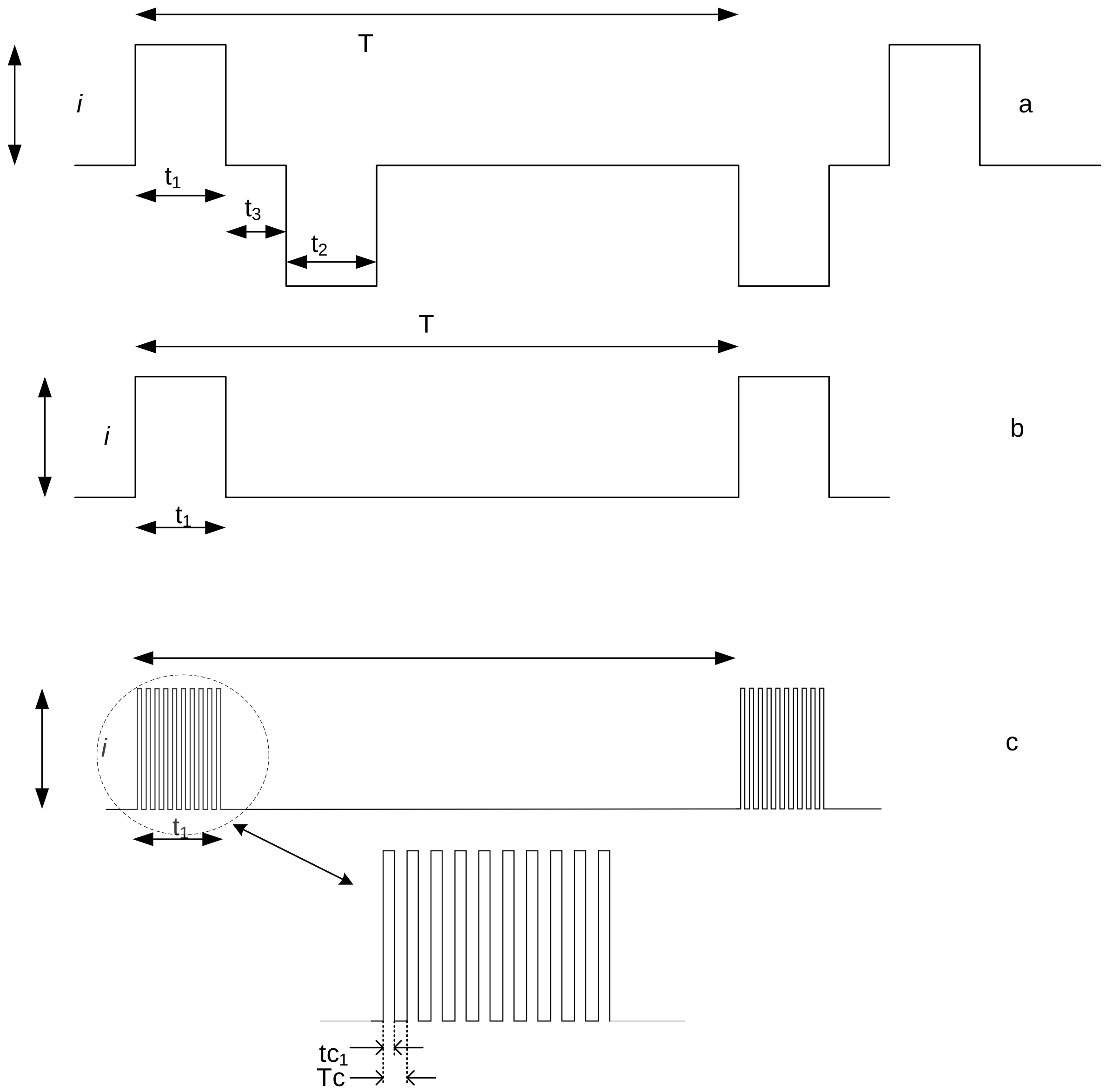

Figure 1. Typical current waveforms used in transcutaneous electrical stimulation. (a) A symmetric biphasic waveform of current amplitude i, with a pulse interval of T, and the two phases having durations of t1 and t1 resp. An interphase interval is shown and is not always present, in which case t3=0. (b) A monophasic waveform. (c) A monophasic waveform where the current pulse is broken into a series of sub-pulses

Figure 2 – PRISMA Flow Diagram

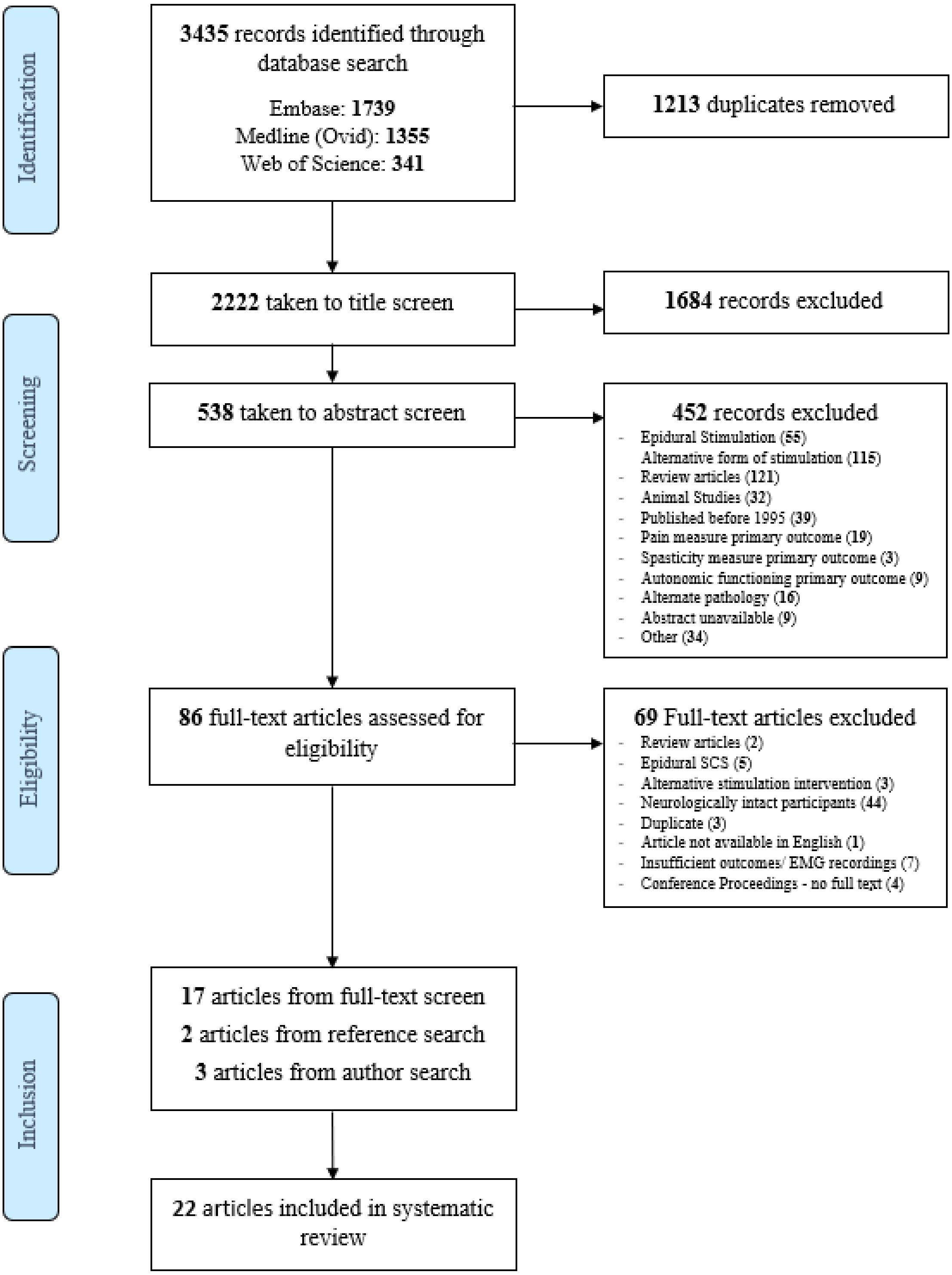

Figure 2. PRISMA Flow Diagram of screening and selection processes

Appendix containing search strategy

Click here to access/download

Supporting Information

S1 Appendix Search Strategy.docx

Click here to access/download
Supporting Information
PRISMA 2009 checklist tSCS and motor response generation.docx
