# Supplementary material for: Transcutaneous spinal cord stimulation and motor responses in individuals with spinal cord injury: A methodological review
Source: PLoS One. 2021 Nov 18;16(11):e0260166. doi: 10.1371/journal.pone.0260166 (PMC8601579; doi:10.1371/journal.pone.0260166)
Supplement: Attachment — Submitted filename: Response to reviewers letter 2.docx [file pone.0260166.s005.docx]

Date: 08/10/2021

Peter Schwenkreis

Editor-in-Chief, Plos One

Subject: # PONE-D-21-17243, Response to Reviewers for the manuscript titled, “**Transcutaneous spinal cord stimulation and motor responses in individuals with spinal cord injury: a methodological review**.”

We thank the reviewers for their critical assessment of our manuscript. We have now revised the second draft and added the two recommended minor changes as per the reviewer’s comments. We thank the reviewers for their time in improving the quality of this manuscript. In the following response we specifically address each point made by the reviewers.

**Comments to the Author**

**1) It would be helpful if the authors are able to provide a reference for the statement "The use of a relatively high frequency carrier waveform in the stimulation pulse is believed to give more efficient signal transfer because the skin-electrode interface impedance has an area-dependent capacitive component which presents less electrical impedance at higher frequency".**

**Response:**

Thank you for highlighting this area as requiring referencing. We have added two references to this statement that detail the capacitance of the skin barrier and the effectiveness of the high frequency carrier.

**2) Given the extended period over which the review was performed, there is an additional article, which I believe should have been included:
Al’joboori, Y., Massey, S.J., Knight, S.L., Donaldson, N. & Duffell, L.D. The Effects of Adding Transcutaneous Spinal Cord Stimulation (tSCS) to Sit-To-Stand Training in People with Spinal Cord Injury: A Pilot Study. Journal of Clinical Medicine, 2020. 9(9): p. 2765.**

**Response:**

Thank you for bringing this article to our attention. Our librarian believes it to have been missed by the search MeSH due to the keywords we included surrounding EMG. This article is now included in the review paper.
